# Annexin A11 mutations are associated with nuclear envelope dysfunction *in vivo* and in human tissues

**DOI:** 10.1093/brain/awae226

**Published:** 2024-07-11

**Authors:** Valentina Marchica, Luca Biasetti, Jodi Barnard, Shujing Li, Nikolas Nikolaou, Matthew P Frosch, Diane E Lucente, Mark Eldaief, Andrew King, Manolis Fanto, Claire Troakes, Corinne Houart, Bradley N Smith

**Affiliations:** Department of Basic and Clinical Neuroscience, Wohl Clinical Neuroscience Institute, Institute of Psychiatry, Psychology and Neuroscience, King’s College London, London SE5 9RX, UK; Centre for Developmental Neurobiology, Institute of Psychiatry, Psychology and Neuroscience, Guy’s Campus, King’s College London, London SE1 1UL, UK; Department of Basic and Clinical Neuroscience, Wohl Clinical Neuroscience Institute, Institute of Psychiatry, Psychology and Neuroscience, King’s College London, London SE5 9RX, UK; Department of Basic and Clinical Neuroscience, Wohl Clinical Neuroscience Institute, Institute of Psychiatry, Psychology and Neuroscience, King’s College London, London SE5 9RX, UK; Department of Basic and Clinical Neuroscience, Wohl Clinical Neuroscience Institute, Institute of Psychiatry, Psychology and Neuroscience, King’s College London, London SE5 9RX, UK; Living Systems Institute, University of Exeter, Stocker Road, Exeter EX4 4QD, UK; Mass General Institute for Neurodegenerative Diseases, B114-2700, Charlestown, MA 02129, USA; C.S. Kubik Laboratory for Neuropathology, Massachusetts General Hospital, Boston, MA 02114, USA; Center for Genomic Medicine, Massachusetts General Hospital, Boston, MA 02114, USA; Mass General Institute for Neurodegenerative Diseases, B114-2700, Charlestown, MA 02129, USA; Department of Basic and Clinical Neuroscience, Wohl Clinical Neuroscience Institute, Institute of Psychiatry, Psychology and Neuroscience, King’s College London, London SE5 9RX, UK; London Neurodegenerative Diseases Brain Bank, SGDP Centre, PO65, Institute of Psychiatry, Psychology and Neuroscience, King’s College London, London SE5 8AF, UK; Department of Basic and Clinical Neuroscience, Wohl Clinical Neuroscience Institute, Institute of Psychiatry, Psychology and Neuroscience, King’s College London, London SE5 9RX, UK; Department of Basic and Clinical Neuroscience, Wohl Clinical Neuroscience Institute, Institute of Psychiatry, Psychology and Neuroscience, King’s College London, London SE5 9RX, UK; London Neurodegenerative Diseases Brain Bank, SGDP Centre, PO65, Institute of Psychiatry, Psychology and Neuroscience, King’s College London, London SE5 8AF, UK; Centre for Developmental Neurobiology, Institute of Psychiatry, Psychology and Neuroscience, Guy’s Campus, King’s College London, London SE1 1UL, UK; Department of Basic and Clinical Neuroscience, Wohl Clinical Neuroscience Institute, Institute of Psychiatry, Psychology and Neuroscience, King’s College London, London SE5 9RX, UK; Centre for Developmental Neurobiology, Institute of Psychiatry, Psychology and Neuroscience, Guy’s Campus, King’s College London, London SE1 1UL, UK

**Keywords:** amyotrophic lateral sclerosis, annexin A11, lamin B2, nuclear envelope

## Abstract

Annexin A11 mutations are a rare cause of amyotrophic lateral sclerosis (ALS), wherein replicated protein variants P36R, G38R, D40G and D40Y are located in a small helix within the long, disordered N-terminus. To elucidate disease mechanisms, we characterized the phenotypes induced by a genetic loss-of-function and by misexpression of G38R and D40G *in vivo*.

Loss of Annexin A11 results in a low-penetrant behavioural phenotype and aberrant axonal morphology in zebrafish homozygous knockout larvae, which is rescued by human wild-type Annexin A11. Both Annexin A11 knockout/down and ALS variants trigger nuclear dysfunction characterized by Lamin B2 mislocalization. The Lamin B2 signature also presented in anterior horn, spinal cord neurons from post-mortem ALS ± frontotemporal dementia patient tissue possessing G38R and D40G protein variants.

These findings suggest mutant Annexin A11 acts as a dominant negative, revealing a potential early nucleopathy highlighting nuclear envelope abnormalities preceding behavioural abnormality in animal models.

## Teaser

Mutations in annexin A11 are a rare cause of amyotrophic lateral sclerosis. Marchica *et al.* show that annexin A11 mutations disrupt the nuclear envelope in animal models in a similar manner to annexin A11 loss-of-function, suggesting that the ALS-causative mutations act via a dominant negative mechanism.

## Introduction

Amyotrophic lateral sclerosis (ALS) is an insidious, late onset neurodegenerative disorder characterized by the deterioration of upper and lower motor neurons. ALS gene therapy trials are underway including tofersen treatment for SOD1 mutation positive patients,^1^ with FDA approval granted in April 2023. However, with the exception of riluzole, no broad effective drug treatments currently exist and there is a mean survival of just 2–5 years post-diagnosis.^2-4^ The underlying causative genetic heterogeneity comprised of mutations in 24 Mendelian genes and a separate complex aetiology coupled with low penetrance (∼90% sporadic inheritance) present challenges for developing translational therapies.^5,6^

Our group recently identified novel or rare protein variants (G38R and D40G) located within a small helix in the N-terminus of the calcium and phospholipid binding gene Annexin A11.^7^ The predominant D40G variant segregated in UK families and post-mortem tissue from a sporadic D40G case had prolific Annexin A11 immunopositive aggregates in spinal cord and hippocampus tissue. Since then, multiple mutation reports from independent, multi-ethnic ALS cohorts have replicated G38R and D40G and identified further novel mutations cementing this mutation cluster—D40I, D40Y and P36R.^8-18^ The spectrum of neurodegenerative phenotypes associated with Annexin A11 mutations now include ALS, frontotemporal dementia (FTD), ALS ± FTD, multisystem proteinopathy, Paget’s disease, muscular dystrophy and oculopharyngeal muscular dystrophy.^9,15,19-21^ This highlights that post-mitotic cell types, such as neurons and skeletal muscle, are particularly vulnerable to mutations in Annexin A11. Experimental large-scale functional investigative analyses have yielded direct associations of Annexin A11 with specific ALS implicated pathways, such as disrupted liquid-liquid phase separation^22^ (LLPS), RNA granule transport via organelles,^23^ stress granules and calcium dynamics.^12,24^ Of note, one study found Annexin A11 to act as a molecular tether, linking RNA granules (N-terminus) with the phospholipid membrane of lysosomes (C-terminus), which are axonally trafficked.^23^

In the present study, we conducted a full characterization of Annexin A11 *in vivo* using *Danio rerio* (zebrafish) and *Drosophila melanogaster* models. We first assessed if Annexin A11 is important to neuronal function by creating a CRISPR/Cas9 knockout of Annexin in zebrafish and longitudinal RNAi knockdown in *Drosophila*. Second, we evaluated the functional effects of replicated ALS N-terminal variants, G38R and D40G in zebrafish larvae. We found that Annexin A11 ALS mutations affect nuclear function and impair Lamin B2 dynamics in a similar way as the loss-of-function (LoF), indicating that the mutant proteins act as dominant negative, preventing the function of the normal alleles. The Lamin B2 signatures found in *in vivo* zebrafish models are also present in spinal cord post-mortem tissue of patients with concordant Annexin A11 variants, signifying a translational relevance of the fish findings to ALS biology.

## Materials and methods

For materials and methods pertaining to *Drosophila*, zebrafish genotyping and antibodies and neuropathology, please see the online Supplementary material.

### Zebrafish study

#### Zebrafish embryos maintenance

Zebrafish (*Danio rerio*) were kept at 28°C with a 14/10-h light/dark cycle. Embryos were obtained from in house breeding of AB strain zebrafish stocks. Starting from 24 hours post-fertilization (hpf), embryos were cultured in fish water containing 0.003% PTU (1-phenyl-2-thiourea; Sigma) to prevent pigmentation and 0.01% methylene blue to prevent fungal growth. The animal experimentations have been authorized by KCL Ethic Review Committee and covered fully by the Project Licence of C.H.

#### Microinjection and misexpression of plasmid constructs in zebrafish embryos

For misexpression studies in AB zebrafish larvae a final concentration of combined UAS:eGFP and MNX1:Gal4 (GIB40_mnx1_5kb-E1b-KalT4-G1) i.e. 20 ng/ul was injected at the one cell stage of fertilized larvae. The UAS promoter of UAS:eGFP (pN2 UAS MCS eGFP) was subcloned to replace the CMV promoter from wild-type and ALS mutant Annexin A11-eGFP plasmids (pANXA11_SGG_SGFP27). Annexin A11-eGFP mutants included G38R, D40G, D40H and AH1del, introduced by mutagenesis (Agilent QuikChange II Site-Directed Mutagenesis Kit, Cat No. #200523). The plasmid mix (20 ng/ul final concentration) was injected into one cell stage AB zebrafish embryos (0.2 nl) using a Picospritzer 111 microinjector (Parker instrumentation). A toxicity gradient ruled out use of 30 ng/μl and 60 ng/μl final concentration, as this was toxic in the Annexin A11 wild-type injected larvae. Uninjected embryos from the same dish were kept as a control for normal development up until 5 days. Immediately after injection, embryos were transferred to a dish containing E3 medium and were incubated at 28°C. Embryos expressing high levels of eGFP at 24 hpf (by observation using a Zeiss UV fluorescent microscope) were selected and taken forward for further analysis.

#### Brightfield imaging

Brightfield images of zebrafish embryos at different developmental stages were taken with a Nikon Digital SMZ1500 Sight DS-Ri1 microscope. A lateral view imaging setup was achieved by placing the embryos on a glass slide with the lateral side facing upwards. Images were taken with a ×10 magnification.

#### *In vivo* analysis of axonal length and branching

Live embryos were mounted in 0.8% agarose in E3 water to investigate the mutant axonogenesis defects by live imaging. Embryos (48 hpf were anaesthetized in 0.02% MS-222 (Tricaine mesylate), embedded in 0.8% low melting point agarose dissolved in E3 fish water and placed in a chamber that was willed with E3 water to allow confocal microscopy (Nikon Eclipse C1 confocal microscope using a 40× water dipping objective). Quantification of caudal primary (CaP) motor axon lengths (measuring the distance where the CaP motor axons extended from the spinal cord past the horizontal myoseptum and towards the most ventral axonal projection) and the number of secondary and tertiary branches were made on confocal stacks, using Fiji Single Neurite Tracer in 12 individual CaPs neuron per variant injected. Any neurons extending shorter than the horizontal myoseptum were excluded from analysis to exclude rostral primary neurons. Imaging or quantification of all larvae at 48 hpf for all experiments was conducted on neurons between somites 7 and 14.

#### Immunohistochemistry

Following microinjection of GFP-tagged DNA or generation of knockout lines of Annexin A11a larvae, positive embryos were fixed with 4% paraformaldehyde (PFA) at 48 hpf. Subsequently, embryos were washed 3 × 5 min in PBS containing 0.8% and then to allow the immunostaining, 1 ml of PBS + 0.8% triton + 0.25% trypsin was added for 22 min. Embryos were then blocked in 10% goat serum and incubated in a primary antibody solution overnight at 4°C, followed by three 2-h washes with PBS + 0.8% triton + 0.25% trypsin. Embryos were then incubated overnight in secondary antibodies at 4°C, followed by 3 × 2 h PBS washes. Embryos were post-fixed with 4% PFA for 20 min at room temperature, followed by three brief PBS + 0.8% Triton washes before being stored in 70% glycerol in the dark.

#### Behaviour

Locomotor behaviour was assessed at 24, 48 hpf and 5 days post-fertilization (dpf). For tail twisting analysis embryos at 24 hpf in a petri dish and recorded for 30 s without any stimulus to access to their spontaneous side-to-side contractions of the trunk and tail. For touch response experiments, 48 hpf larvae were lightly touched on the tail with a needle. The embryos were placed at the centre of a 150 mm diameter petri dish filled with E3 fish water and scored from 0 to 4 [0: no movement, 1: flicker of movement (slight movement) but no swimming, 2: movement away from probe but with impaired swimming, 3: normal swimming].

#### Acridine orange staining

Apoptotic cells were identified in 48 hpf living embryos by adding 5 mg/ml acridine orange vital dye (N-4638; Sigma) to the E3 medium (5 mM NaCl, 0.17 mM KCl, 0.33 mM CaCl_2_, 0.33 mM MgSO_4_). Following that, embryos were incubated in the dark at 28°C for 30 min, washed 5× quickly in E3, anaesthetized and viewed under Zeiss confocal microscope. Apoptotic cells were identified in 2-day-old living embryos by adding 5 mg/ml acridine orange vital dye (N-4638; Sigma) to the embryo water. Following that, embryos were incubated in the dark at 28°C for 1.5–2 h, washed, anaesthetized and viewed under a microscope.

#### mRNA rescue experiments

The mutant rescue was performed by injecting N-terminal T7 promoter mRNA generated wild-type Annexin A11-eGFP and D40G-Annexin A11-eGFP [Annexin A11-eGFP on a pCDNA3.1(+) backbone] into one-cell stage embryos. Annexin A11-eGFP mRNA was generated using the mMessage T7 kit (Ambion, Cat. No. AM1344). Rescue was assessed at 24 and 48 hpf by assessing spinal cord phenotype, motor neuron expression and behaviour. A minimum of 390 ng/embryo was used for phenotype rescue experiments.

#### Live imaging and confocal microscopy

Live embryos were mounted in 0.8% agarose in E3 water to investigate the expression of each microinjected plasmid. Embryos (48 hpf) were anaesthetized in 0.02% MS-222 (Tricaine mesylate), embedded in 0.8% LMP agarose dissolved in E3 fish water and imaged using a Nikon Eclipse C1 confocal microscope using a 40× water dipping objective, and/or Zeiss LSM 800 Confocal using a 20× water dipping objective. Imaged embryos were fixed in 4% PFA overnight for immunostaining. Fluorescent triple immunostained embryos were captured on the Zeiss LSM 800 Confocal with Airyscan using a 40× and 63× lens.

### Behavioural tests

#### Spontaneous coiling

Groups of 10 dechorionated embryos were arrayed in a petri dish and analysed under a Zeiss Stereo Discovery V20 microscope. Three-minute recordings were obtained with a Zeiss AxioCam camera [at a frame rate of 30 frames per second (fps)]. Movies were obtained in .avi format and converted to .mp4. Videos were played back in slow motion using QuickTime Player (Apple) and coiling or tail flicking events for each embryo was manually counted.

#### Touch evoked escape response

Individual embryos (48 hpf) were placed in a petri dish and mounted on Zeiss Stereo Discovery V20 microscope. Embryos were left without any stimuli for 5 min and then touched lightly with a pipette tip to elicit the escape response. Recordings were obtained with a Zeiss AxioCam camera at a frame rate of 30 fps. The swim path of their escape response was recorded and analysed using the following scoring system: 0, no movement; 1, flicker of movement (slight) but no swimming; 2, movement away from probe but with impaired swimming; 3:, normal swimming.

#### Optokinetic response

Saccades movement was tested using a custom-built behavioural recording system at 5 dpf. The device comprised a plastic drum lined with alternating black and white stripes, a stereoscope equipped with a light source connected to a CCD camera, and a variable speed motor connected to a belt that rotates the drum. In a 35 mm plastic dish, a 5 dpf single larva was mounted in an upright position in 3% methylcellulose solution. The dish was positioned in the rotating drum’s centre. Each larva was screened and recorded for 5 min without stimuli and then using a series of clockwise revolution spins of the drum at a speed of 10 rpm. Individual larvae videos were watched to determine their response to the rotating drum. Optokinetic response (OKR) responsive larvae followed the rotating stripes and adapted to variations in the direction of the turning drum, whereas OKR non-responsive larvae did not respond to the rotating drum.

## Results

### Loss of Annexin A11 impairs motor and visual functions in animal models

**Figure 1:**
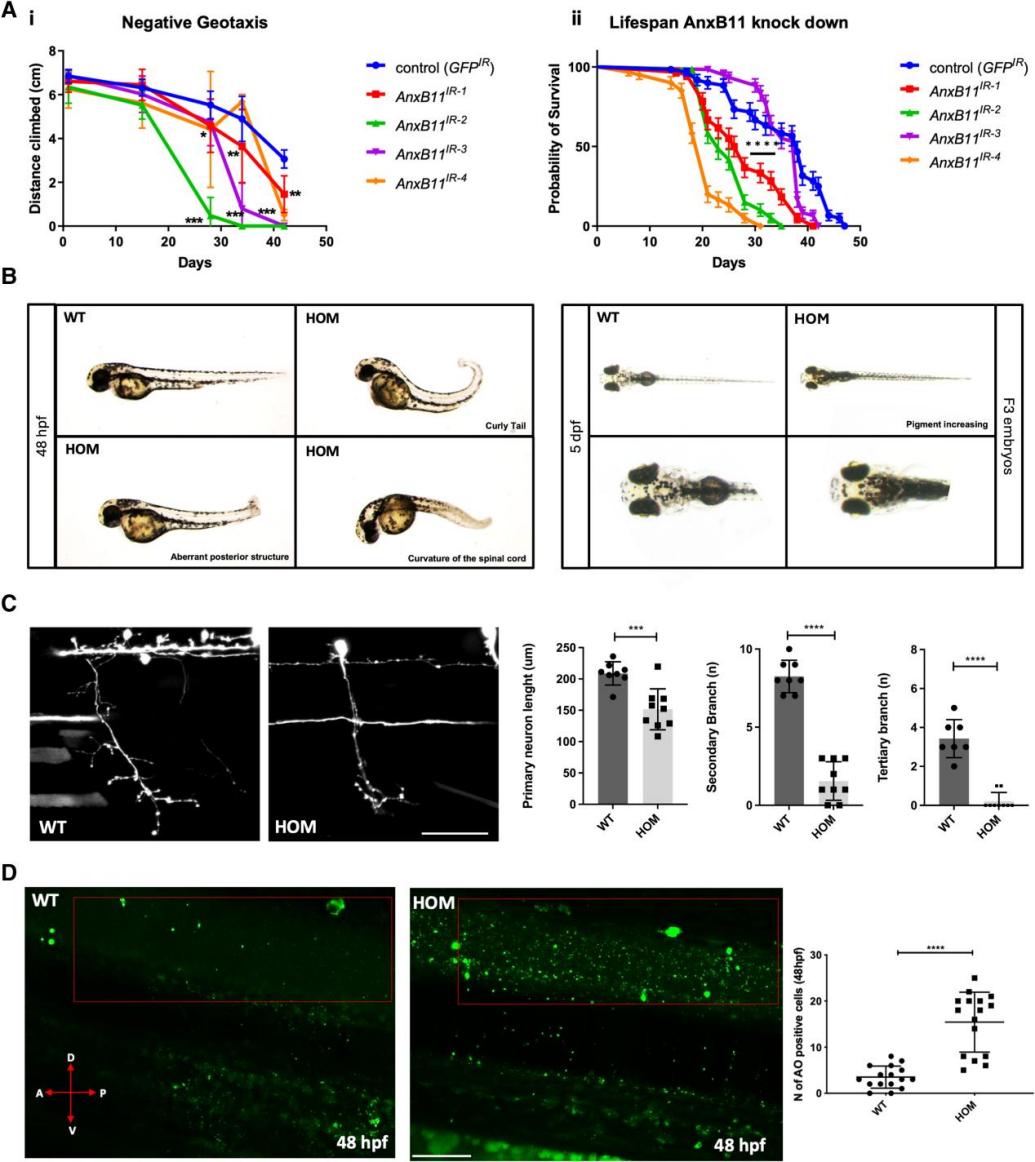
**Loss of Annexin A11 function in *Drosophila* and zebrafish result in behavioural and neuronal phenotypes.** [**A**(**i**)] Knockdown of *Drosophila* AnxB11 with four siRNAs [KK101313 (IR-1), GD36186 (IR-2), GD36185 (IR-3) and GD29693 (IR-4)] and a control RNAi against GFP specifically in glutamatergic neurons, including motor neurons in the fly, with the OK371-Gal4 driver identified a time-dependent climbing phenotype after 28 days under all treatments. Two-way ANOVA, Sidak’s multiple comparison for each age *P ≤ 0.05, **P ≤ 0.01, ***P ≤ 0.001. [**A**(**ii**)] When driven in all adult neurons with the Elav-Gal4 driver and the temperature-dependent repressor Ubi-Gal80ts three out of four AnxB11 knock down determined a significant shortening in fly lifespan with respect to control flies). Kaplan-Meyer Log Rank test ****P ≤ 0.0001. (**B**) CRISPR-Cas9 knockout of zebrafish Annexin A11a results in a low penetrant, pleiotropic phenotype observed in ∼30% of F3 homozygous larvae characterized by spinal defects (*left*), with ∼20% of larvae showing an increase of skin pigment at 5 dpf (days post-fertilization) implicating ocular defects (*right*). (**C**) Loss of function leads to abnormalities in axonal length and branching via live imaging of caudal primary (CaP) motor neurons in wild-type (WT) and homozygous (HOM) larvae of primary, secondary and tertiary branches at 48 hpf (hours post-fertilization). Quantitative analysis of axonal length of primary, secondary and tertiary branches after microinjection of eGFP empty vector in WT and HOM identifies significant loss of branching, *post hoc* Tukey’s multiple comparisons test; axon length ***P ≤ 0.005, secondary and tertiary branches ****P ≤ 0.0001. *n* = 8 WT, *n* = 9 hom. Scale bar = 100 μm. (**D**) Acridine orange (AO) staining of 48 hpf larvae shows a significant increase in apoptotic cells (green). WT and HOM panels showing lateral view and red boxed region of spinal cord for quantification of apoptotic cells. Quantification of number of AO-positive cells in WT and HOM larvae. Expression and cell count data were similar in each replicate (19 cells ± 1.117, 24 hpf; 15 cells ± 1.734 48 hpf) compared to WT siblings (14 cells ±1.117, **P ≤ 0.005 24 hpf; 4 ± 1.734 ****P ≤ 0.0001, 48 hpf; unpaired *t*-test). Scale bar = 50 μm.

Annexin A11 is expressed in most tissues during development and in adults.^25^ To assess the contribution of Annexin A11 to neuronal architecture and function we either knocked down or out the corresponding orthologues in *Drosophila* and zebrafish, respectively. The *Drosophila* orthologue, AnxB11 has a high C-terminus but limited N-terminal conservation to human Annexin A11 in most AnxB11 transcripts. The zebrafish orthologue, Annexin A11a, is 67% conserved with human Annexin A11, with all residues mutated in ALS patients from our initial publication conserved in zebrafish. Furthermore, Annexin A11a contains the conserved motif, PIGLDN, predicted to form the first of two predicted amphipathic helices (AH1), also found in human Annexins A1 and A2,^25^ and absent from the zebrafish paralogue Annexin A11b. An overview of zebrafish Annexin A11a and b orthologues can be seen in Supplementary Fig. 1. A panel of four RNAi lines (refer to the ‘Materials and methods’ section) was used to knockdown the fly Annexin A11 orthologue. To assess any locomotor differences, we expressed all RNAi lines in glutamatergic neurons, which includes motor neurons in *Drosophila* and conducted a negative geotaxis assay.^26^ In this assay we detected a significant loss of climbing ability with age in all RNAi lines (Fig. 1A). Correspondingly, when the AnxB11 RNAi lines were driven in all neurons, specifically in the adult fly, there was also a significant deficit in survival in a lifespan assay^26^ with three out of four lines progressing with further impairment at 30 and 40 days (Fig. 1B). In parallel, we characterized an in-house CRISPR/Cas9-mediated Annexin A11a knockout zebrafish carrying a premature termination codon in exon 4 (Y197fs21X) referred to as Annexin A11 knockout (AKO). F1 knockout individuals were in-crossed, to create an F2 generation of wild-type, heterozygous and homozygous adults. Most homozygous individuals are viable. All experiments were comparisons between wild-type (WT), heterozygous (het) or homozygous (hom) mutant F3 embryos and larvae (see Supplementary material regarding the generation of lines). To determine if the AKO leads to a loss of Annexin A11 mRNA, we conducted qPCR, which showed a significant reduction of Annexin A11a mRNA, with homozygous 2 dpf embryos showing a 10-fold transcript decrease (Supplementary Fig. 2). Morphological analysis of F3 individuals at 2 dpf showed that ∼30% of homozygous larvae display a range of morphological spinal cord defects compared to wild-type (Fig. 1B, 70% look morphologically normal and grow into viable adults). At 5 dpf, ∼20% of normal-looking homozygous larvae display an increased pigmentation of the skin. Like many aquatic vertebrates, zebrafish larvae adjust their melanin skin pigment distribution in response to ambient light levels and increased pigment in melanocytes often indicates visual impairment.^27^ An OKR test confirmed a significant visual impairment (refer to the ‘Behaviour analysis’ section).

### Loss of Annexin A11 disrupts neuronal architecture

To assess morphology of motor neurons, we analysed the zebrafish ventrally innervating primary spinal motor neurons (CaP) in wild-type siblings and normal-looking homozygous larvae using live imaging. Embryos were injected with a UAS:GFP construct driven by mnx1:Gal4 at the 1-cell stage to mosaically label motor neurons. At 2 dpf, GFP-positive CaP motor neuron axons extend ventrally from cell bodies in the spinal cord to innervate ventral muscles. Quantification of total motor axon length and number of branches in wild-type and homozygous larvae (Fig. 1C) revealed significantly shorter and less branched axons in mutant compared to wild-type (WT = 8; Hom = 9; *P* ≤ 0.005). In addition to developmental abnormalities in neuronal maturation, loss of Annexin A11 increases neuronal cell death. Indeed, acridine orange staining at 24 and 48 hpf in mutants with normal gross morphology both showed a significant increase in apoptotic cells at 48 hpf (Fig. 1D, **P ≤ 0.005 24 hpf and ****P ≤ 0.0001, 48 hpf; unpaired *t*-test).

#### Human Annexin A11 restores the zebrafish null’s synaptic architecture and motor function

**Figure 2:**
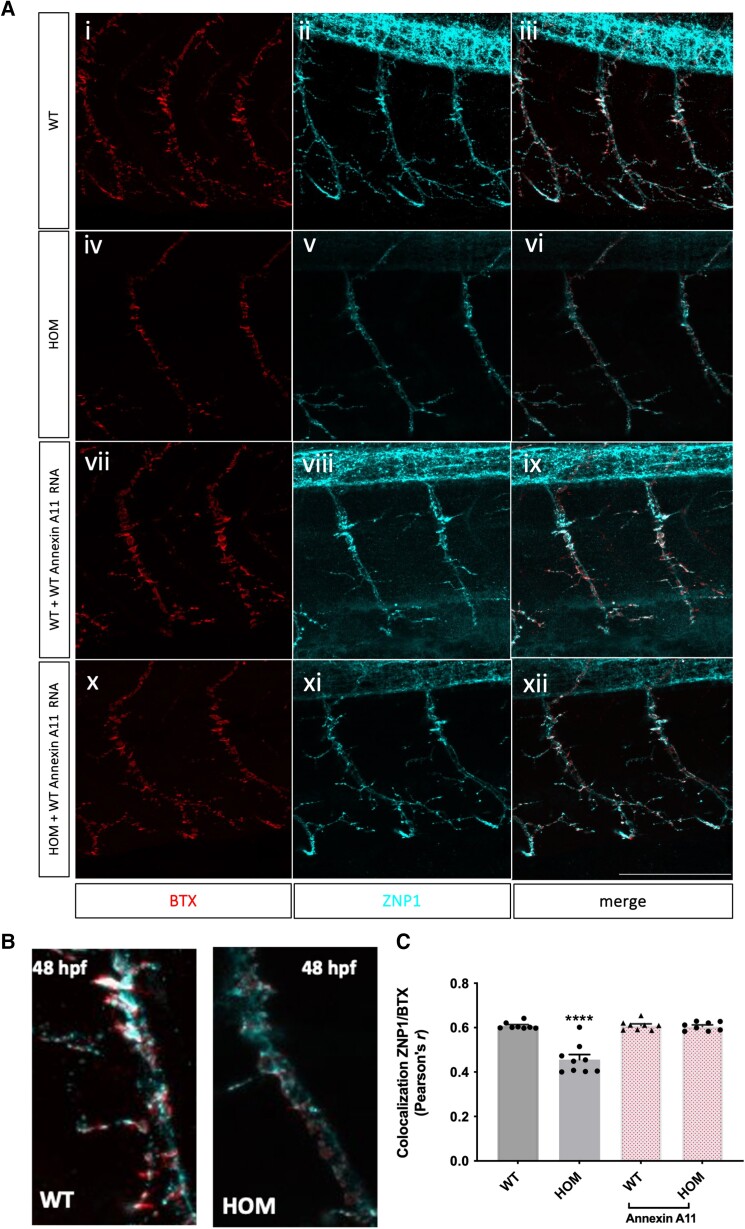
**Loss of function of Annexin11a results in defects of zebrafish neuromuscular junctions**. (**A**) Fixed wild-type (WT) and homozygous (HOM) larvae were stained to label pre and postsynaptic junctions with ZNP1 (blue), αBTX (red) and merge respectively with representative images shown. HOM larvae [(**iv–vi**), *n* = 9 embryos] show a loss of branching reflected by a loss of Znp1/Btx co-localization compared to WT [**A**(**i–iii**) (*n* = 8 embryos) and in **B**]. This was significant on quantification (**C**) (Pearson’s *r* coefficients of co-localization, *P* ≤ 0.0001). Injection with human WT Annexin A11 mRNA had no detrimental effect on the WT larvae [**A**(**vii–ix**) (*n* = 8 embryos)]. Homozygous embryos injected with human WT Annexin A11 (*n* = 9 embryos) rescued the loss of ZNP1 and αBTX co-localization [**A**(**x–xii**)] that was significant (**C**, unpaired *t*-test; ****P ≤ 0.0001). Max projections, ×40 oil objective. Scale bar = 100μm. hpf = hours post-fertilization.

As homozygous larvae display reduced elongation and branching, we investigated any corresponding synaptic consequences. We therefore examined the pre- and post-synapses of the neuromuscular junction (NMJ) in both wild-type and homozygous normal-looking embryos by endogenous staining with the pre- and postsynaptic markers ZNP-1 (vesicular) (synaptotagmin-2) and α-BTX (α-Bungarotoxin), respectively. Confocal imaging and analysis of neurons within axonal somites 7–14 at 48 hpf show aberrant neuronal morphology and a lack of pre and postsynaptic connections in homozygous AKO larvae compared to wild-type [Fig. 2A(i–vi)]. These NMJ defects were rescued by injection of human Annexin A11 mRNA at the one-cell stage as homozygous larvae showed a restoration of pre- and postsynaptic co-localization and axonal branching at 48 hpf [Fig. 2A(vii–xii), B and C]. This demonstrates the conservation of function of Annexin A11 from fish to humans and its key role in maintenance and function of motor neurons.

In addition, we conducted standard behavioural assays to assess if disrupted neuronal morphology and neuromuscular connectivity were associated with motor behaviour. We first assessed spontaneous tail twisting, which begins at the 15-somite stage as extending primary motor neurons form the first NMJs.^28^ In homozygous knockout larvae, the number of spontaneous coils per minute were significantly reduced compared to wild-type larvae (*P* ≤ 0.0001, Supplementary Videos 1 and 2). Injection of human wild-type Annexin A11 mRNA fully rescued this reduction, confirming re-establishment of neuronal architecture (Supplementary Videos 3 and 4 and Supplementary Fig. 3). At 48 hpf, the touch evoked escape response test (TEER) was employed to assess the engagement of fast twitch muscles that are innervated by primary motor neurons.^29^ After touch stimulus to larval tails, a strong swimming response occurred in wild-type larvae but was severely reduced in normal-looking homozygous larvae that showed no movement nor a flicker (slight) response (*P* ≤ 0.0001) (Supplementary Fig. 4 and Supplementary Videos 5 and 6). Similarly to the spontaneous coiling, injection of human wild-type Annexin A11 restored the knockout’s capacity to respond (Supplementary Video 7) and wild-type larvae injected with human wild-type Annexin A11 show normal escape response (Supplementary Video 8). This demonstrates that Annexin A11 is critical to the development and function of motor circuitry.

Having observed a build-up of melanin in 5 dpf larvae in the knockout of Annexin A11a we used the OKR to assess if larvae presented with moving objects across their vision field have a loss or gain of saccadic eye movements.^30^ Homozygous larvae had a significantly reduced degree of saccadic eye movements compared to wild-type larvae. These results suggest that although visual impairments can lead to a reduction in saccadic eye movements, the decrease of saccades seen in the KOs could also indicate a reduction of oculo-motor activity. Similarly, this phenotype was rescued by injection of human Annexin A11 mRNA at the one-cell stage (Supplementary Fig. 5 and Supplementary Video 9).

Our behavioural analysis confirms a pleiotropic phenotype due to loss of Annexin A11a that presents with significant penetrance. Both neuronal morphology and motor and visual behaviour can be restored by human Annexin A11.

### Misexpression of ALS-associated Annexin A11 mutations alter its subcellular localization in motor neurons

**Figure 3:**
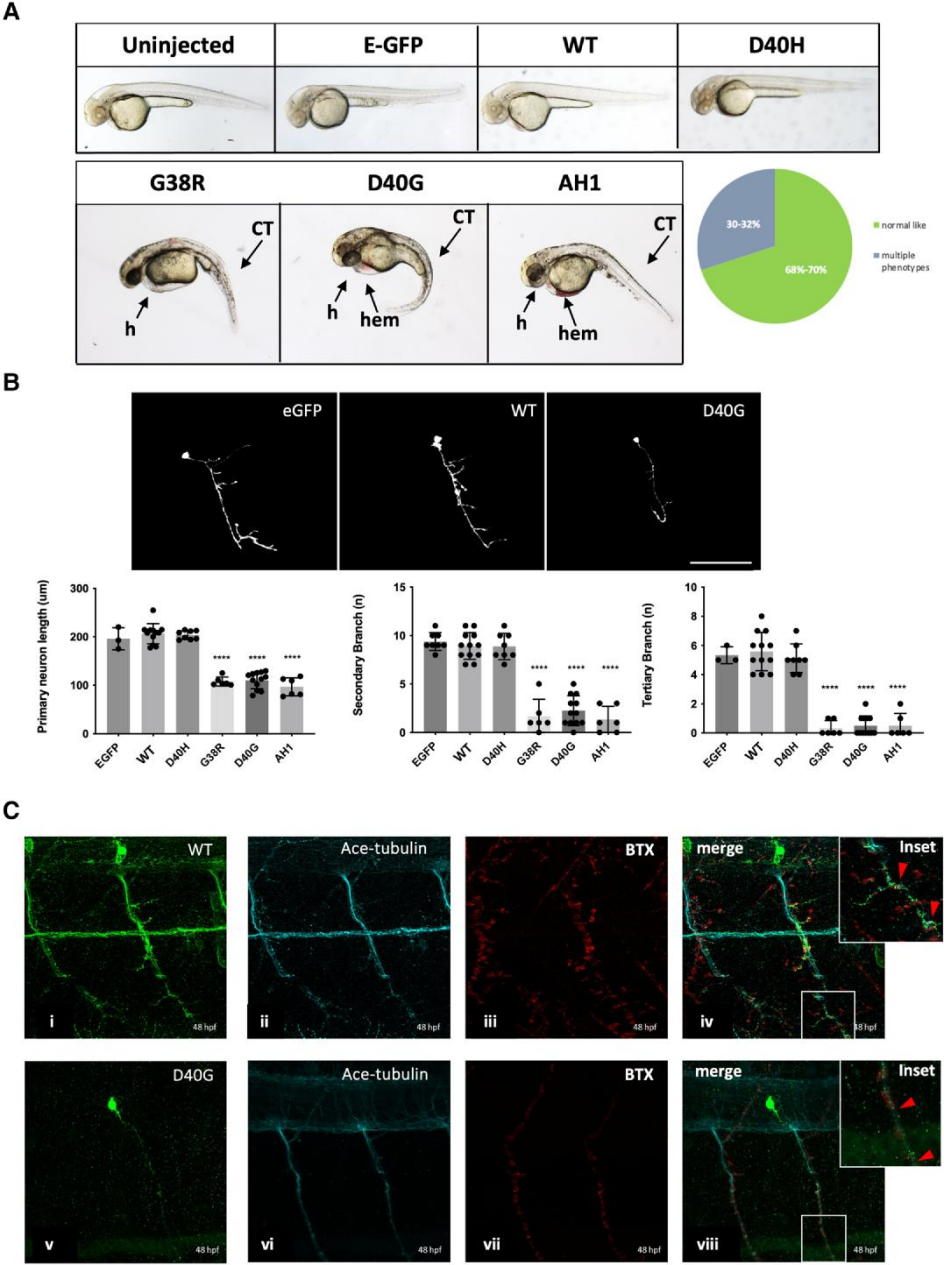
**Misexpression of p.D40G, p.G38R and AH1-Annexin A11 in AB Zebrafish result in severe morphological defects at 48 hpf.** (**A**) Lateral view of AB larvae injected at the one-cell stage expressing MNX1:GAL4 and a mix of either empty-UAS GFP, or human Annexin A11-GFP plasmids of wild-type (WT), the non-disease control D40H and mutants D40G, G38R and AH1 (alpha helical 1 deletion) and imaged at 48 hours post-fertilization (hpf). Approximately 30% of larvae with G38R, D40G and AH1 mutants showed a range of morphological phenotypes i.e. curly tail (CT), haemorrhage (hem) and heart defects (h) (*n* = 40 each group, *n* = 12 G38R, D40G and AH1 deformed embryos each time, four biological replicates). (**B**) Live imaging of caudal primary (CaP) motor neurons from normal like GFP positive (+ve) larvae (free of morphological abnormalities) showing representative images (*left* to *right*) injected with empty vector-eGFP, wild-type Annexin A11-eGFP and D40G-Annexin A11-eGFP. Neurons imaged from sagittal and cross-sectional view show a single neuron innvervated by a cap neuron (*top*). Quantitative analysis of axonal length and branching after microinjection with control eGFP vector, wild-type Annexin A11 and amyotrophic lateral sclerosis (ALS) and AH1 mutants (*bottom*). Statistical analysis was performed on GraphPad Prism 7 using a one-way ANOVA with a *post hoc* Tukey’s multiple comparisons test. ****P ≤ 0.0001, *n* = 3: eGFP: *n* = 12 WT-Annexin A11-eGFP, *n* = 12: D40G- *n* = 6 G38R and *n* = 6 AH1 Annexin A11-eGFP (one axon per biological replicate, maximum projection). Scale bar = 100μm. (**C**) The D40G mutation results in a reduced amount of axonal Annexin A11 expression and clustering with acetylcholine receptors. [**C**(**i–viii**)] Confocal images of zebrafish trunk, lateral view, anterior to the right, imaged at 48 hours post-fertilization (hpf) after microinjection with WT-Annexin A11-eGFP and D40G-Annexin A11-eGFP mutant plasmids and followed by GFP [**C**(**i** and **v**)], ac-Tubulin [**C**(**ii** and **vi**, cyan)] and staining BTX [**C**(**iii** and **vii**, red)]. GFP immunohistochemistry (IHC) demonstrates decreased or absent branching of motor axons in D40G injected embryos, compared to embryos injected with WT-Annexin A11. *Insets* on [**C**(**iv** and **viii**)] represent effects on postsynaptic development (red arrows and white square). Max projections. Scale bar = 100μm. eGFP = enhanced green fluorescent protein.

**Figure 4:**
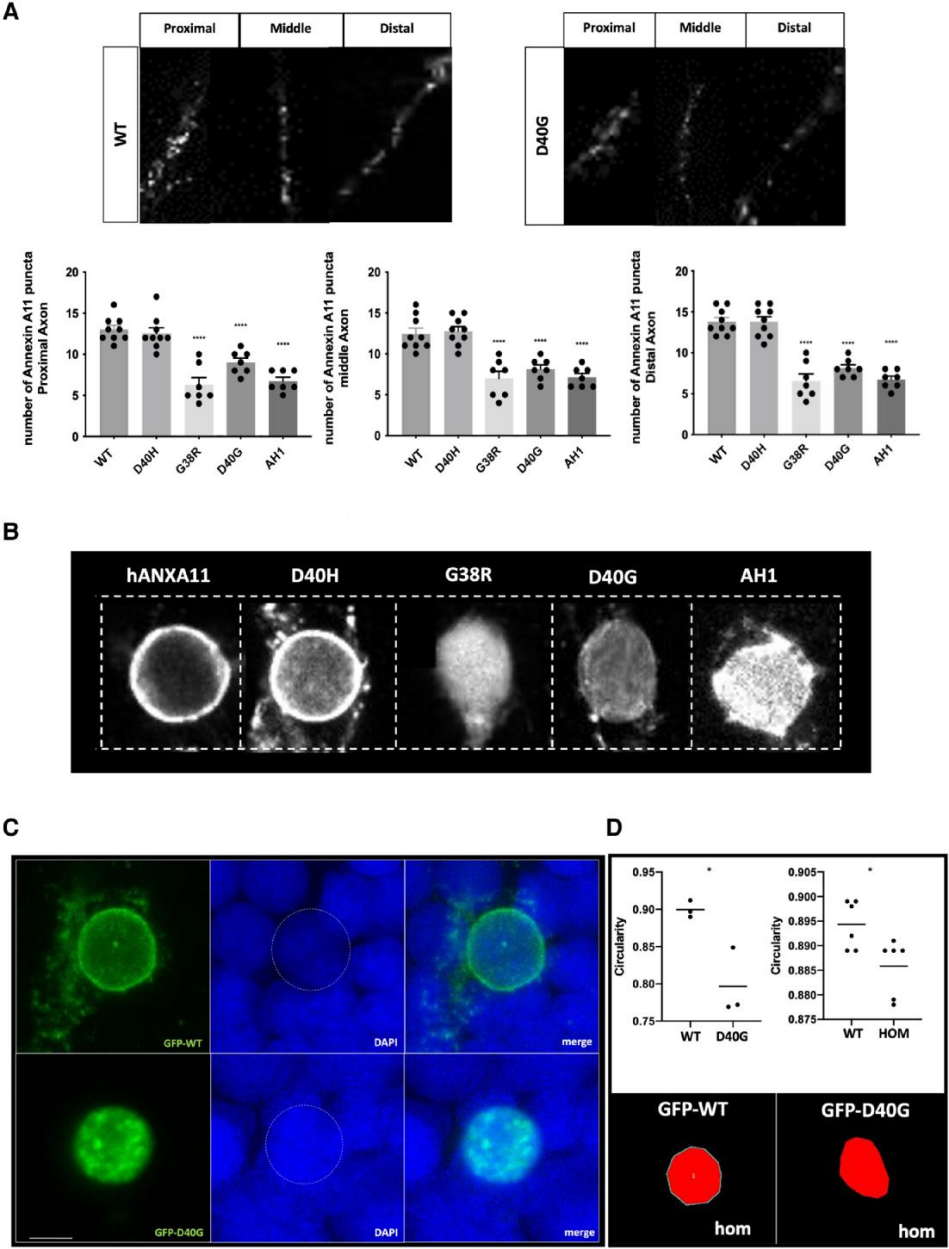
**WT-Annexin A11 is present as puncta throughout the axon and is reduced due to ALS or AH1 del mutations.** (**A**) Representative single plane images of wild-type (WT) and D40G Annexin A11 positive puncta in the proximal, middle and distal axon segment (*top*). Puncta were quantified in axons from larvae injected with controls WT Annexin A11, D40H and mutants D40G, G38R and AH1 del. Total *n* caudal primary (CaP) neurons = 39 (one per embryos): WT-Annexin A11-eGFP (*n* = 9), D40H-Annexin A11-eGFP (*n* = 9), G38R-Annexin A11-eGFP (*n* = 7), D40G-Annexin A11-eGFP (*n* = 7) and AH1-Annexin A11-eGFP (*n* = 7) (*bottom*). All three mutations reduce the amount of Annexin A11 puncta in each segment. ****P ≤ 0.0001, one-way ANOVA, Tukey’s multiple comparison test. (**B**) Annexin A11 mutations alter Annexin A11 nuclear envelope distribution. Qualitative GFP immunofluorescence shown *left* to *right*, wild-type, D40H, G38R, D40G and AH1del-Annexin A11-eGFP and counterstained with DAPI (signal not shown). Images are ×63 and ×3 digital zoom (Zeiss LSM 800). (**C**) Misexpression of human WT Annexin A11 can rescue nuclear envelope integrity in homozygous Annexin A11a knockout larvae. *Top* (*left* to *right*): Wild-type Annexin A11-eGFP when expressing in knockout Annexin A11a larvae localizes to the nuclear envelope and as perinuclear puncta (eGFP and DAPI merge). *Bottom* (*left* to *right*): D40G Annexin A11-eGFP by contrast is completely nucleoplasmic with the presence of intra-nuclear aggregates (eGFP and DAPI merge). Scale bar = 5 μm. (**D**) Nuclear circularity changes are associated with loss of Annexin A11a or the D40G mutation in Annexin A11. *Bottom*: Representative images from circularity analysis of homozygous knockout larvae expressing wild-type or D40G Annexin A11-GFP. Quantification (*top left*) shows a significant deviation from circularity. *P ≤ 0.05, unpaired *t*-test wild-type (*n* = 3) and D40G (*n* = 3). Similarly, F3 homozygous knockout larvae (*top right*) show a deviation of nuclear circularity from F3 wild-type larvae of the same line, unpaired *t*-test *P ≤ 0.05 [WT (*n* = 6) and homozygous (Hom, *n* = 6)]. ALS = amyotrophic lateral sclerosis; GFP = green fluorescent protein.

To evaluate the impact of ALS-associated variants on Annexin A11 localization and behaviour, we mosaically expressed eGFP-tagged wild-type, G38R and D40G Annexin A11 in motor neurons (injection of mnx1:gal4; UAS:AnxA11-GFP at the one-cell stage). Also included were a construct harbouring an in-frame deletion of the first predicted α-helical domain (AH1, residues 36–41 i.e. PIGLDN) and a control non-disease related polymorphism, D40H (rs368751524). At 48 h, ALS and AH1 variants presented with severe developmental deformities comprised of heart defects, haematomas and a curvy tail (due to leakiness of the mnx1:gal4 expression in non-neuronal cells, Fig. 3A). Larvae expressing wild-type or control constructs were phenotypically identical to uninjected larvae. Live confocal imaging of GFP positive motor neurons at 48 h showed that expression of G38R, D40G and AH1 variants induced morphological defects comprised of shortening of axons and loss of secondary and tertiary branches (*P* ≤ 0.0001) [Fig. 3B(i and ii)]. Immunostaining and confocal imaging demonstrated that WT-Annexin A11-GFP was present both in the nucleus and as puncta in the axon, and distally with synapses [Fig. 3C(i–iv)]. In addition to loss of branching, the D40G variant at a qualitative level also displayed an apparent loss of axonal Annexin A11 [Fig. 3C(v–viii)]. We therefore examined wild-type human Annexin A11 and all mutants in both axonal and nuclear compartments. In an axonal analysis we quantified the Annexin A11 pool of puncta within the proximal, middle and distal segments of mosaically expressing axons. G38R, D40G and the AH1 variants showed a significant reduction in axonal puncta compared to wild-type and D40H (Fig. 4A). To examine distal synapses, we conducted immunohistochemistry (IHC) on fixed 48 hpf larvae mosaically expressing wild-type and D40G Annexin A11 with synaptotagmin-2 (ZNP-1, presynaptic marker) and α-BTX (NMJ) (Supplementary Fig. 6). Extended motor neurons expressing wild-type Annexin A11-eGFP indicated a co-localization between presynaptic ZNP-1 vesicles and α-BTX. The D40G mutation significantly altered this co-localization and reduced motor neuron innervation patterns at the NMJ, most probably due to a decreased capability in forming secondary and tertiary branches (Supplementary Fig. 7A and B). The loss of co-localization of pre- and postsynaptic markers in the Annexin A11 ALS variant larvae mirrors what we observed in our homozygous knockout larvae at the same age (Fig. 3). Therefore, both loss of Annexin A11 and N-terminal ALS-associated variants lead to an aberrant axonal structure and perturbed axonal function contributing to degeneration of early post-mitotic neurons.

Closer inspection of the nuclear compartment (Supplementary Fig. 6) found abundant nucleoplasmic Annexin A11 and intranuclear aggregates in D40G expressing neurons (Supplementary Fig. 6E). This contrasted to wild-type Annexin A11 that localized to the nuclear envelope as well as low level nucleoplasmic staining (Supplementary Fig. 6A). We repeated mosaic expression in wild-type as well as all mutants, focussing on the nucleus and observed that all ALS variant alleles conferred the same nucleoplasmic signature (Fig. 4B).

In the zebrafish Annexin A11 knockout, we rescued morphological and axonal architecture/function with human wild-type Annexin A11 mRNA (Fig. 2). To further assess if the nuclear phenotype could be rescued, we mosaically expressed human wild-type and D40G Annexin A11 eGFP in Annexin A11a homozygous knockout larvae at the one-cell stage. Fixing, IHC and imaging at 48 hpf identified that human wild-type Annexin A11 highlighted the same localization pattern as mosaic expression in non-transgenic larvae (Fig. 4C) i.e. predominantly nuclear envelope and puncta in the soma. The D40G variant shows purely nucleoplasmic Annexin A11 and evidence of intranuclear inclusions. Furthermore, we replicated the behavioural phenotype and toxicity observed in the mosaic DNA injections of D40G Annexin A11 by injecting D40G Annexin A11 mRNA into the homozygous Annexin A11a knockout (Supplementary Fig. 8). Last, we noticed that nuclei of mosaically expressing Annexin A11 D40G and other mutants were irregular in shape (Fig 4B and C). Circularity analysis identified a significant shift from normal circularity in the mosaic D40G larvae and additionally, quantification of nuclei from non-mosaic homozygous knockout spinal cord identified the same deviation from circularity compared to wild-type (Fig. 4D). We conclude from these experiments that Annexin A11a is required for maintenance of nuclear architecture and that ALS mutant proteins likely repress the nuclear function of the normal Annexin A11, leading to a toxic dominant interference with nuclear organization in motor neurons.

### Loss of Annexin A11 impacts Lamin B2 localization

**Figure 5:**
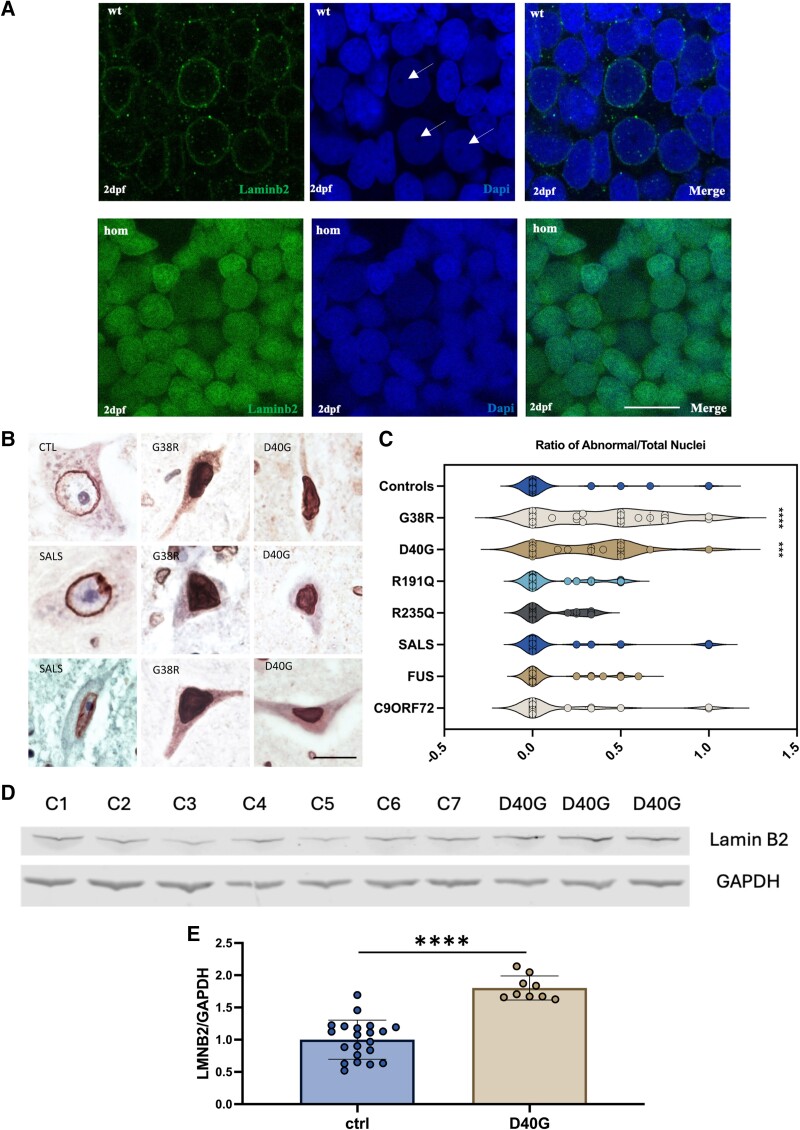
**Lamin B2 localization shifts from the nuclear envelope to the nucleoplasm in Annexin A11a homozygous larvae at 48** **hpf and post-mortem tissue from patients with Annexin A11 mutations**. (**A**) *Top*: Endogenous Lamin B2 (green) in wild-type (WT) Annexin A11a larvae is localized to the nuclear envelope (and in *merge*). Large nucleoli are indicated by white arrows (DAPI). *Bottom*: In Annexin A11a homozygous knockout larvae a pool of Lamin B2 shifts to the nucleoplasm (green and DAPI merge) with a loss of large nucleoli (DAPI). (*n* = 3) Scale bar = 20 uM. (**B**) Brightfield Lamin B2 staining of individual anterior horn neurons revealing preserved nuclear membrane staining in the control (CTL) and sporadic ALS (SALS) cases, but diffuse nuclear staining in the G38R and D40G Annexin A11 mutation cases. Scale bar = 50 µm. (**C**) Violin plot quantifying the mean ratio of the number of neurons containing abnormal nucleoplasmic LMNB2 compared to total number of neuronal nuclei per field (×40 magnification). This included controls (*n* = 9), G38R, D40G, R191Q (*n* = 4) non-disease polymorphism, with a minor allele frequency (MAF) of 4.7% in Europeans, R235Q, SALS (*n* = 5), FUS positive cases (*n* = 6) and cases with the C9ORF72 GGGGCC expansion (*n* = 2). Only the G38R and D40G cases showed a significant association with nucleoplasmic LMNB2 (G38R versus Controls, ****P ≤ 0.0001; D40G versus Controls, ***P ≤ 0.005; Kruskal-Wallis test for multiple comparisons). (**D**) Western blot Lamin B2 expression of post-mortem motor cortex tissue from an Annexin A11 D40G patient (*n* = 3) and control cases (*n* = 7) compared to GAPDH expression. (**E**) Quantification of Lamin B2 expression in D40G tissue is ∼1.8-fold higher compared to controls (****P ≤ 0.0001, unpaired *t*-test). dpf = days post-fertilization; Hom = homozygous.

As nuclear distribution of Annexin11 is perturbed by ALS-linked mutations in zebrafish and Annexin A11 normally localized in the nuclear envelope, we assessed whether the nuclear envelope is regulated by Annexin 11a. Owing to the elongated, oval shape of spinal cord nuclei expressing the G38R and D40G variants, we probed for endogenous Lamin B2. A previous study by Coffinier *et al*.^31^ found cortical neuron nuclei of LMNB2 knockout mice to be elongated and oval shaped, that matched our observations in ALS-variant zebrafish. We examined the distribution of endogenous Lamin B2 (LMNB2) in our Annexin A11a wild-type and homozygous knockout individuals. Confocal slices from fixed 48 hpf larval spinal cords display a classic nuclear envelope localization in wild-type. However, there was a striking shift of a pool of LMNB2 from the nuclear envelope to the nucleoplasm, with a loss of large nucleoli in the mutant lacking Annexin A11a (Fig. 5A). This suggests a key functional relationship between Annexin A11 and Lamin B2 that is essential for nuclear integrity. A change in nuclear lamina could influence the internal nuclear and chromatin architecture including DNA stability and transcription, and potentially impair transport through the nuclear pore.^32^ When the sole endogenous Lamin B type protein localization was examined in AnxB11 knock-down flies in glutamatergic neurons, we noticed a loss of lamina circularity, which implies defects in nuclear integrity, as previously shown^33^ and a partial Lamin disruption at 22 days of age (just before onset of climbing phenotype) as seen in fish larvae (unpaired *t*-test *P* < 0.0001, Supplementary Fig. 9). We speculate that partial Lamin disruption in flies at 22 days may be due to the smaller and variable length Annexin A11 N-terminus compared to zebrafish and humans.

### Nucleoplasmic LMNB2 extends to post-mortem patient tissue with Annexin A11 variants

Our finding of nuclear dysfunction in misexpressed Annexin ALS variants and Annexin A11a knockout studies in fish larvae, has been conducted in neurons ∼28 h after exiting the mitotic cycle.^34^ We wished to explore whether this signature was translated to mature end stage spinal cord motor neurons of ALS patients harbouring dominant associated Annexin A11 missense variants. We matched those ALS variants examined in fish misexpression studies with immunostaining of post-mortem spinal cord tissue sections from an unpublished sporadic ALS/FTD case harbouring a G38R variant and our original sporadic ALS case with a D40G variant.^7^ Clinical details of the G38R case can be found in the Supplementary material. The D40G case has previously been described in detail^7^ to possess Annexin A11 and TDP43 pathology. Similarly, Annexin A11 3,3'-diaminobenzidine (DAB) staining of the G38R (ALS/FTD case) showed very occasional skein-like annexin A11 immunopositive neuronal cytoplasmic inclusions (NCIs) in the spinal cord of the G38R case, together with abundant NCIs and neurites in the motor cortex (Supplementary Fig. 10). We also stained spinal cord tissue accessed from a previously identified sporadic ALS case, which had an Annexin A11 R235Q variant^7^ (located in the first Annexin A11 domain). The R235Q case displayed no evidence of Annexin A11 positive inclusions (Supplementary Fig. 11). This suggests that the R235Q variants may be a private polymorphism or a susceptibility factor acting via another disrupted mechanism of Annexin A11. With the confirmation of Annexin A11 immunoreactive aggregates in our G38R and D40G cases, we specifically investigated large neurons of spinal cord anterior horn for LMNB2 pathology. Interestingly, DAB immunostaining with LMNB2 of the spinal cord showed a high proportion of nucleoplasmic LMNB2 in G38R and D40G cases, as seen in spinal cord CaP neurons of Annexin A11a homozygous knockout fish (Fig. 5B and Supplementary Fig. 12). To quantify this phenomenon, we determined the ratio of neurons with fully nucleoplasmic LMNB2 nuclei/total stained nuclei, in sequential ×40 magnification field views of spinal cord anterior horns from our cases with either G38R, D40G or R235Q variants. We compared this to counts from nine healthy controls, five sporadic ALS (sALS) and a further four controls possessing an N-terminal p.R191Q Annexin A11 polymorphism (present in 4.7% age matched controls, rs2229554) to account for common Annexin A11 variation in the background population. Staining also included spinal cord sections from ALS cases harbouring FUS variants (*n* = 6) and C9ORF72 GGGGCC expansion ALS cases (*n* = 2). We observed that neurons of the anterior horn in G38R and D40G cases had a higher proportion of nucleoplasmic Lamin B2 when compared to controls and cases with either FUS variants or C9ORF72 GGGGCC expansions (both ****P ≤ 0.0001) (Fig. 5C). However, the R235Q case was not significantly different from controls. Examining further cases with the R235Q variant or other Annexin A11 domain variants are required to validate this preliminary finding but these initial findings may imply that the N-terminus is specific to Lamin B2-Annexin A11 function and maintaining nuclear envelope integrity. Interestingly, a previous study of changes to nuclear lamina invaginations using Lamin B1 in post-mortem motor cortex from C9ORF72 cases did not reveal any pathological association with ALS.^35^ Even though we did not investigate nuclear invaginations in our study, we similarly did not detect an association of nucleoplasmic Lamin B2 of spinal cord anterior horn cells in C9ORF72 cases. This suggests potentially an absence of Lamin B2-associated nuclear envelope dysfunction in C9ORF72 cases, but rather perhaps more specific nuclear pore nucleocytoplasmic trafficking issues, as previously outlined.^36^ However, Lamin B2 expression in our Annexin A11 KO fish and DAB staining of patient tissue qualitatively increased. We therefore quantified Lamin B2 expression by western blot from motor cortex of the D40G patient used in this study; and identified a significant ∼1.8 increase in Lamin B2 expression (****P ≤ 0.0001; Fig. 5D and E and Supplementary Fig. 13). Further studies are required to corroborate this finding, however, it points to conceivable Lamin B2 misregulation in the nuclear compartment, potentially altering chromatin structure. A recent study found that Lamin B2 was highly expressed in non-small cell lung cancer cells (NSCLC) promoting the upregulation of H3K9me2, which is linked to transcriptional repression.^37^

### Lamin B2 signature is present in motor cortex and spinal cord of G38R Annexin A11

**Figure 6:**
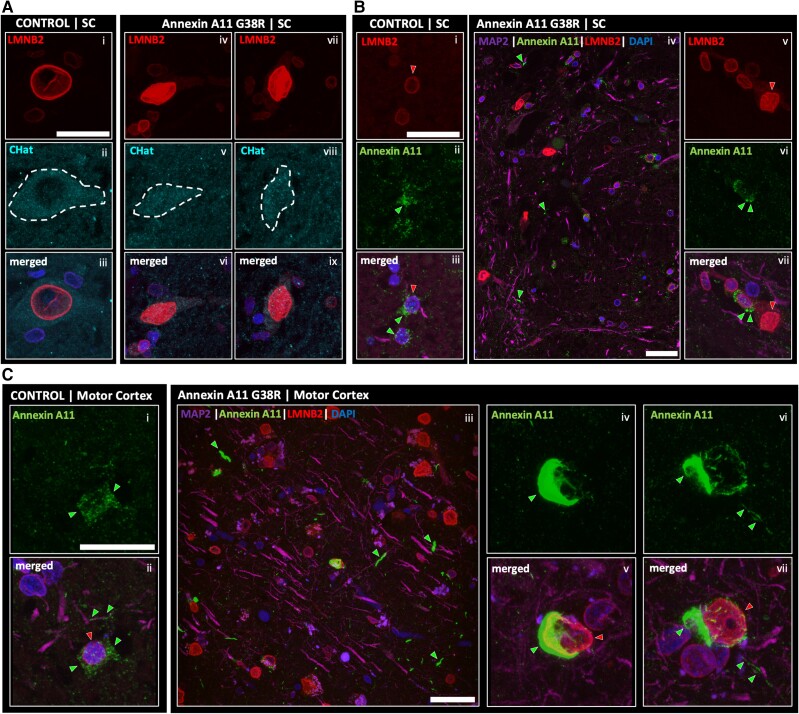
**Immunostaining of Lamin B2 and Annexin A11 in controls and Annexin A11 mutant ALS+/FTD cases.** (**A**) Immunostaining of LMNB2 in a representative control and spinal cord (SC) of anterior horn neurons from the G38R case. Tissue was stained for LMNB2, ChAT (choline acetyltransferase) to label motor neurons and DAPI. A representative ChAT positive motor neuron [**A**(**ii**)] displaying a nuclear envelope localized LMNB2 signal [**A**(**ii** and **iii**)]. Two ChAT positive motor neurons [**A**(**v** and **viii**)] from the G38R case showing abnormal nucleoplasmic LMNB2 [**A**(**iv**, **vi** and **vii**, **ix**), respectively]. Images presented as 3D *z*-stack projections. Scale bar = 50 µm. (**B**) Immunostaining of a representative control and spinal cord of anterior horn neurons from the G38R case displaying the difference in Annexin signal. Wild-type ring-like LMNB2 [**B**(**i**), red arrow] and cytoplasmic diffuse Annexin signal [**B**(**ii**), green arrow] in a representative control neuron [**B**(**iii**)]. Images presented as 3D *z*-stack projections. Single plane image from the anterior horn region of spinal cord tissue from the G38R case [**B**(**iv**)]. Neurite aggregates of Annexin can be seen (green arrows). Representative image of neurons reiterating the LMNB2 nucleoplasmic signal [**B**(**vi** and **vii**, red arrow)] and displaying a more punctate Annexin signal [**B**(**vi** and **vii**, green arrows)]. Images presented as 3D *z*-stack projections. Scale bar = 50 µm. (**C**) Immunostaining of a representative control and motor cortex neurons from the G38R case showing a marked presence of Annexin aggregation. Representative control motor cortex neuron displaying a diffuse cytoplasmic Annexin signal [**C**(**i** and **ii**)]. Motor cortex from the G38R case showing nucleoplasimc LMNB2 signal and neuropil Annexin aggregates (green arrows) [**C**(**iii**)]. Neurons from the G38R case motor cortex reporting heavy perinuclear Annexin aggregates [**C**(**iv**, **v** and **vi**, **vii**), respectively; green arrows for Annexin aggregates, red arrows for LMNB2 nucleoplasmic signal]. Images presented as 3D *z*-stack projections. Scale bar = 50 µm.

To complement our LMNB2 DAB study, we conducted Annexin A11 and LMNB2 immunofluorescence staining on human post-mortem spinal cord from our G38R case alongside controls (Fig. 6A and B). Motor neuron staining with ChAT (choline acetyltransferase) and LMNB2 in the anterior horn of spinal cord showed predominant LMNB2 staining in the nuclear envelope of control nuclei [Fig. 6A(i–iii)]. However, the G38R case showed the concordant LMNB2 nucleoplasmic signature seen by DAB staining [Fig. 6A(iv–ix)]. In control cases, Annexin A11 appeared as small puncta located in neuronal soma, especially concentrated in the perinuclear region, with small pools of puncta present in the nuclear envelope and in axons [Fig. 6B(i–iii)]. In the G38R case, single plane wide-angle imaging revealed nucleoplasmic LMNB2 and Annexin A11 positive aggregates in neurites [Fig. 6B(iv)], with an abundance of large perinuclear Annexin A11 aggregates and a loss of smaller puncta [Fig. 6B(iv–vii)]. In our original DAB staining, we did not observe obvious nucleoplasmic LMNB2 in motor cortex neurons in ALS mutant cases (data not shown). Therefore, we conducted immunofluorescence imaging of motor cortex to determine the extent of the LMNB2 signature. After analysis, like spinal cord, Annexin A11 puncta in control was abundant in the soma of neurons but also present in axons (MAP2 staining) of control tissue with LMNB2 located in the nuclear envelope [Fig. 6C(i and ii)]. Wide-view plane imaging of G38R motor cortex showed an abundance of Annexin A11-positive neuronal torpedos in the neuropil and nucleoplasmic LMNB2 staining of nuclei [Fig. 6C(iii)]. Cortical axonal Annexin A11 inclusions were very large, encapsulating the nucleus with multiple smaller, filament-like inclusions emanating outwards, including aggregate structures within the axon [Fig. 6C(iv–vii)]. This highlights a significant putative impairment in axonal and nuclear function in both motor cortex and spinal cord. Compromised nuclear envelope function due to N-terminal missense Annexin A11 mutations may represent a lowly penetrant, early, upstream disease mechanism impairing Lamin B2 dynamics with a putative impact on nucleocytoplasmic transport and axonal health.

## Discussion

Our functional investigation of Annexin A11 ALS variants (G38R and D40G) in zebrafish and human post-mortem tissue, and knockout/knock-down models in zebrafish and *Drosophila* converge on a toxic, disruptive effect on nuclear envelope function that correlates with a loss of synaptic function. First, ∼25% of homozygous AKO zebrafish display low-penetrant spinal cord deformities, including a loss of saccadic eye movements suggesting disruption to movement of ocular and axial motor neurons, with vision potentially affected. A recent report identified a childhood onset *de novo* p.D40I Annexin A11 variant in a patient with oculopharyngeal muscular dystrophy, suggesting pleiotropic effects.^19^ Interestingly, ocular abnormalities, including significant alterations to saccadic movement, have been identified in ALS patients in multiple studies.^38,39^ Second, we found that knockdown of AnxB11 in *Drosophila* developed an age-dependent climbing phenotype. Therefore, this demonstrates that Annexin A11 is important for neuronal function.

Our models point to a relationship of Annexin A11 with Lamin B2 and a key role in maintaining nuclear envelope function. Annexin A11 may provide structural support in anchoring Lamin B2 to the nuclear phospholipid membrane (either directly or indirectly) or alternatively regulating Lamin B2 function. Interestingly, Annexins 4 and 5 are also known to associate with the nuclear envelope.^40-42^

Alterations in Lamin B2 localization and expression could have consequences for nucleoskeletal architecture affecting chromatin organization, transcription, epigenetic control and—coupled with the ageing process—may act as a general neurodegenerative risk factor.^43^ This is supported by our preliminary finding that Lamin B2 expression is upregulated in motor cortex of the D40G patient. Furthermore, our post-mortem staining shows a correlation of Annexin A11 pathology with nucleoplasmic Lamin B2. Though limited in the number of mutations studied, both G38R and D40G display Annexin A11 pathology and nucleoplasmic Lamin B2, however R235Q tissue was absent for both pathologies. The specificity of the nucleoplasmic Lamin B2 signature may therefore be exclusively linked to Annexin A11 aggregation and to what degree this dysfunction is present in non-genetic mediated Annexin A11 ALS remains to be established.

In our Annexin A11 over-expression zebrafish model, we did observe an enrichment of wild-type Annexin A11 in the nuclear envelope, as opposed to pools of Annexin A11 puncta in axonal soma, the nuclear envelope and axons in post-mortem control tissue from adult individuals. This may be due to differences in age or time-dependent expression and localization of Annexin A11. However, LMNB2 mislocalization and disruption was conserved across the Annexin A11 animal models (knockdown, knockout or expression of ALS missense changes) and in post-mortem tissue from ALS patients harbouring N-terminal Annexin A11 variants. Even though zebrafish misexpression of ALS variants resulted in nuclear accumulation, the aggregation of G38R Annexin A11 was mirrored in human motor cortex, which displayed unique large aggregates encapsulating the nuclear envelope. This has potential consequences as nuclear-cytoplasmic transport may be compromised with TDP43 shuttling and function particularly impaired as ALS patients carrying either G38R or D40G mutations have TDP43 pathology.^7,15^ The large nuclear sheathed Annexin A11 aggregates in motor cortex including prolific neuronal torpedoes from the G38R case suggest that cortical neurons may be more vulnerable to Annexin A11 dysfunction.

An early study found that Annexin A11 has a role in mitosis at the nuclear envelope, potentially mediated by the N-terminus at early and late prophase (nuclear envelope breakdown) and in late telophase (nuclear envelope reformation) before returning to the nucleoplasm.^44^ In zebrafish, the G38R and D40G ALS variants are statically nucleoplasmic (even with *in vivo* calcium fluctuations) most probably due to misfolding and disrupted liquid phase disruption leading to aggregation.^12,45^ Furthermore, in our Annexin A11 knockout, Lamin B2 was also nucleoplasmic, further suggesting a plausible role for Annexin A11 in nuclear envelope reformation. Regardless of any putative mitotic disruption, the LMNB2 defect is present in zebrafish post-mitotic motor neurons as well as neurons from post-mortem tissue of Annexin A11 ALS patients. This dysfunction of the nuclear pool of Annexin A11 may represent an early, upstream event acting as an ALS susceptibility factor. Furthermore, our observation of a significant reduction of Annexin A11-positive puncta in zebrafish mutant neurons, suggests that ALS variants may result in instability, formation of aggregates and degradation. The AH1 deletion mutant supports this as knockout of a portion of the helical region would invariably disrupt Annexin A11 phase separation, as previously described.^12,23^ This loss of functional axonal Annexin A11 could also negatively impact motor neuron development, potentially by a reduced trafficking of RNA granules to distal axons resulting in impaired local translation, leading to NMJ dieback.^23,46-48^ Annexin A11 aggregates may also sequester/destabilize other RNA binding proteins (RBPs), further altering RNA metabolism.^12^

We have identified that LMNB2 mislocalization is associated with nuclear dysfunction with elevated overall expression, however it is plausible that mutated or loss of Annexin A11 may also affect axonal LMNB2 fidelity. Recently, it was identified that axonal LMNB2 localized to mitochondria in cultured *Xenopus* retinal ganglion cells, in which LMNB2 knockdown disrupted mitochondrial morphology, reduced mitochondrial membrane potential and also impaired lysosome trafficking.^49^ Investigating potential dysfunction of axonal LMNB2 in Annexin A11-mediated ALS is scope for future studies. Similarly, axonal Annexin A11 was recently found to act as a tether linking RNA granules (N-terminus) to lysosomes (phospholipid binding C-terminus) in the recent work by Liao and colleagues.^23^ It was found that C-terminal Annexin A11 ALS associated variants break the tether that joins RNA granules to lysosomes. In contrast, D40G impeded RNA granule trafficking to distal axons, but did not significantly alter RNA granule:lysosome tethering. This suggests that N- and C-terminal Annexin A11 ALS variants may act by different disease mechanisms or tether severing may be mutation-specific. Also, further work specifically clarifying the effect of Annexin A11 dysfunction on overall nuclear architecture and pore structure i.e. the behaviour of other Lamin family members, such as Lamin B1 (also proposed to have a role in nucleophagy^33^), is necessary to identify if and how nuclear dysfunction is linked to axonal changes that contribute to degeneration.

Of note, most Annexins repair damaged membranes,^50^ but also display neurotrophic/neuroprotective effects by reducing inflammation and Annexins 1, 2 and 5 have been found to repair tissue damage in spinal cord injury.^51-53^ Our rescue experiments in the AKO line demonstrates that Annexin A11 is necessary for neuronal/synaptic function, including a potential role in structural maintenance of the distal neuron. This suggests that therapeutic application of Annexin A11 (or Annexins) may be viable as a general neurotrophic factor.^54,55^ Our data show a synergistic relationship between Annexin A11 and Lamin B2 to plausibly maintain nuclear envelope dynamics. Potentially Annexin A11 may also have a role in nuclear membrane repair. Annexin A5 has been shown to anchor negatively charged lipids by inducing a phase transition within the underlying lipid bilayer, providing a stable platform for membrane resealing.^56^ Similarly, Annexin A11 may behave in a similar manner with a dual role in RNA and lipid phase transitioning in both the axonal and nuclear compartments.^22^ Phase transitioning driven by the N-terminus of Annexin A11 has recently been proposed to induce a coupled phase state of negatively charged phospholipids in the lysosomal membrane, regulated by known interactors ALG2 and calcyclin.^57^ ALG2 also has a reported role in plasma membrane repair in a complex with the ESCRT (endosomal sorting complex required for transport) complex, therefore Annexin A11 with ALG2 may participate together in nuclear resealing.^58,59^ In light of findings from a recent structural study,^60^ identifying neuronal-specific Annexin A11 interactors may yield key mechanistic insight into clarifying potential coupled phase state relationships for all membrane classes in neurons.

A limitation of our study is a lack of cell biology, but future exploration of induced pluripotent stem cell (iPSC) Annexin A11 models in particular, will provide insight into mechanisms of perturbed nuclear envelope dynamics. In addition, investigation of longitudinal Annexin A11 mutant animal models will determine when Lamin B2 displacement occurs and its impact on the disease process.

In summary, our data suggest that N-terminal Annexin A11 mutations may represent a dominant negative, early upstream event leading to nuclear envelope defects that contribute to ALS pathology.

## Supplementary Material

awae226_Supplementary_Data

## Data Availability

All reasonable requests for data will be made available by contacting the corresponding author.
