# Supplementary material for: Annexin A11 mutations are associated with nuclear envelope dysfunction in vivo and in human tissues
Source: Brain. 2024 Jul 11;148(1):276–90. doi: 10.1093/brain/awae226 (PMC11706284; doi:10.1093/brain/awae226)
Supplement: awae226_Supplementary_Data [file awae226_supplementary_data.zip › Supplementary_Information.pdf]

Supplementary Information

Generation of Annexin A11a Knockout Zebrafish Line

Exon 4 (ENSDART00000101775.5) in Zebrafish of the Annexin A11 orthologue Annexin A11a (ENSDARG00000077383), was targeted for knockout, as the conserved N-terminal alpha-helix (PIGDLN) was conserved in both humans and Zebrafish. Additionally, all other residues mutated in Annexin A11 in ALS patients were conserved in Annexin A11a. The second Annexin A11 zebrafish orthologue, Annexin 11b, did not contain these conserved elements. A high cutting efficiency CRISPR site was detected and targeted for cleavage (c.574_592), and CRISPR primers designed with ZiFit (http://zifit.partners.org). 100uM of each CRISPR primer was annealed and cloned in-between two BsaI restriction sites of pDR274 immediately upstream of a T7 promoter site. After incorporation and plasmid linearized, RNA was made using a T7 promoter primed MEGAshortscript kit from Ambion (AM1354). 1ul of 40ng/µl of guide RNA was co-injected with 1µl of Cas9 RNA (180ng/µl) at the 1 cell stage of 100 AB embryos to create double-strand breaks. At 48hrs, a random selection of 16 embryos from the entire clutch were DNA extracted, and CRISPR/Cas9 efficiency assessed (which was ~70%) using High Resolution Melt Assays (HRMA) on an Applied Biosystems ViiATM 7 Real-Time polymerase chain reaction System (RT-PCR). The remaining clutch were placed in nursery to grow to adult state (3 months). Founder F0 fish were crossed with wild-type AB fish to make F1 progeny. Sequencing of Annexin A11a Exon 4 from 24 random F1 larvae identified the p.Y197fs21X mutation to be the most common frame-shift change, and this was chosen to take forward to downstream experiments. Outcrossed adult F1 p.Y197fs21X larvae were in-crossed to make an F2 generation. An F3 larval generation was made from F2 for all behavioural and cellular experiments in the study. This comprised WT, heterozygous or homozygous fry aged up to 5dpf.

PCR Primers – Zebrafish Studies

Application	Primer	Sequence
Genotyping	ANXA11A-4F	CACCAGCTGTCTCCCCTAACCAGCC
	ANXA11A-4RA	CCAGTTCATAATATGGCCATTGG
qPCR	ANXA11A-NT-F	GACAGCCAGGGCAATATCCT
	ANXA11A-NT-R	ACCATCGGCAGCAAACTGAT
	ANXA11A-CT-F	TCAAGAACACACCGGCCTAC
	ANXA11A-CT-F	CTTCAGAGCGGGTCACCATT

Clinical and Neuropathological Information G38R Patient

The clinical history in this 63-year-old man was that of progressive behavioural changes with anxiety and depression for approximately 6 years. This was followed by motor, speech and swallowing deficits consistent with a diagnosis of FTD-ALS. The histology from the spinal cord showed loss of anterior horn cells with scattered p-TDP-43 immunopositive neuronal cytoplasmic inclusions. There was also loss of myelin in the lateral corticospinal tracts. The motor cortex showed evidence of neuronal loss with gliosis and numerous p-TDP-43 positive neuronal cytoplasmic inclusions (NCIs) and neurites. The Annexin A11 immunohistochemistry (Annexin A11) showed very occasional positive NCIs in the cord. There were numerous ANXA11 immunopositive NCIs and neurites in the motor cortex. These were in a similar pattern to the p-TDP-43 pathology. Overall, this appears pathologically to support the diagnosis of FTLD-ALS.

Supplementary Methods

Drosophila Study

Drosophila Maintenance and Husbandry

All fly stocks were routinely maintained at 18°C in an incubator with a standard 12-hour light cycle on a standard fly food mixture of yeast, agar, and cornmeal with nipagin and propionic acid. For aging experiments, desired progeny were maintained at 29°C and 60% humidity with a 12-hour light cycle.

Drosophila Genotypes

The following Drosophila stocks were used: OK371-Gal4, UAS-CD8-GFP, UAS,GFPIR, UASAnxB11IR-KK101313, UASAnxB11IR-GD29693, UASAnxB11IR-GD36185, UASAnxB11IR-GD36186.

Drosophila Climbing Assays

Fruit flies (Drosophila melanogaster) were selected and aged at 29°C. Age-matched female flies of the indicated genotypes were placed into empty 70mm tubes in groups of 10 flies. When flies are tapped to the bottom of a vial, they immediately climb back to the top of the vial due to their innate negative geotaxis abilities. To address motor function, flies were tapped to the bottom of the vial, and we counted the number of centimeters climbed by the flies in 2-minute intervals over 5 trials. The number of flies climbing to each cm increment was scored, and a genotype average was calculated for each time point across the 5 trials. Flies that jumped or did not carry out a vertical climb in one movement burst were excluded from that trial.

Drosophila Immunohistochemistry

For all immunofluorescence results shown, adult female fly brains were dissected at 22 days of aging at 29°C just prior to the end stage of the lifespan. Drosophila AnxB11 knockdown (GD29693 RNAi line) and w1118 as a control using the OK371-GAL4 driver and the UAS-CD8-GFP transgene to label the affected neurons with GFP. OK371-GAL4 drives the expression of UAS-containing transgenic constructs in glutamatergic neurons, including motor neurons in Drosophila. Adult flies were aged at 29°C for 22 days. Female flies were dissected, and whole brains were incubated with an anti-GFP polyclonal Rabbit antibody (Invitrogen, #A-11122) at a dilution of 1:200 and anti-lamin Dm0 Mouse antibody (DSHB, #ADL67.10) at a dilution of 1:500 for immunostaining. Brains were imaged on the confocal inverted Nikon A1R and the nuclear circularity in GFP positive cells was analysed (NIS Elements, circularity analysis).

Zebrafish Genotyping, RT-qPCR, Antibodies, Statistics and Nuclear Circularity

DNA extraction and genotyping of Annexin A11 CRISPR mutant

After collection of 4 mm square of tail fin from each anesthetized adult fish (using MS222), tissue was digested overnight with DNA extraction buffer (100mM tris/Hcl, 200mM KCl and 5mM EDTA) + 0.1 mg/ml of proteinase K at 55oC. The next morning, proteinase K was neutralised by heating to 80°C for 45mins. 1ul of DNA was then used as template for a genotyping PCR.

Primer design and Sanger sequencing

Primers were designed by using Primer3 (http://bioinfo.ut.ee/primer3). Annexin A11a X4 PCR products amplified from Annexin A11a knockout larvae were directly sequenced by Sanger sequencing. Sequencing of PCR products and plasmids was outsourced to Source BioScience (http://www.sourcebioscience.com/) to identify genotype status.

RT-qPCR

Quantitative PCR (qPCR) was conducted using a light cycler 96 (Roche) qPCR machine. Total RNA was extracted from 10 pooled 48hpf embryos from WT embryos and p.197fs21X larvae heterozygous and homozygous for the mutation. cDNA was made using 500ng of RNA as template. qPCR was conducted using two specific primer sets, the first for the N-terminus of Annexin A11 and the second for the C-terminus. Beta actin was used as a control house-keeping gene.

Antibodies used in Zebrafish Studies

All antibody details used in Zebrafish studies are listed below, with their corresponding dilutions, manufacturer and catalogue number.

Primary and Secondary Antibody	Species	Dilution for Immunostaining	Manufacturer	Catalogue Number
Anti-acetylated tubulin	Mouse	1:500	Sigma	T7451
Anti-GFP	Rabbit	1:100	Amsbio	TP401
α-Bungarotoxin 555		1:100	Thermoscientific	B35451
Znp-1	Mouse	1:200	Abcam	AB154035
Lamin B2	Mouse	1:100	Abcam	AB8983
Anti-mCherry AB	Rabbit	1:500	Abcam	AB167453
DAPI		1:1000	Invitrogen	D1306
Alexa anti-mouse	Goat anti-mouse	1:500	Thermoscientific	A11001
Alexa anti-rabbit	Goat anti-rabbit	1:500	Thermoscientific	A11008
Anti-rabbit 633	Goat anti-rabbit	1:100	Thermoscientific	A21050

Analysis and statistics

All images were processed on ImageJ/Fiji (Version 2.0) software programs and were used for quantitative analysis. According to the nature of the study, single plan or maximum projection images were analysed. Analysis was performed by merging each channel and then Cap motor neurons were manually traced. Statistical analysis was performed on GraphPad Prism 8 using a One-Way ANOVA multiple comparisons test or student t-test.

Nuclear circularity

Nuclear circularity was assessed using FIJI Version 2.0) based on DAPI and LaminB2 or WT Annexin A11 and p.D40G. Larvae were imaged with the Zeiss Airyscan 880 microscope at 63x and raw images were processed with Airyscan Processing. Fiji software generating outlines of nuclei, enabling measurement of nuclear envelope circularity using Analyse Particles function in Fiji, from a maximum projection in order to retain all the signal from all the slices. Particles were analysed with size set to >100µm, circularity >0.3. Data from Fiji was analysed in GraphPad Prism for statistical analysis.

Neuropathology

Patients

ALS Patients harbouring the Annexin A11 D40G and R235Q mutations were sourced from the London Neurodegenerative Diseases Brain Bank (King's College London, U.K). The tissues were from 10% formalin-fixed, paraffin-embedded tissue blocks and full consent for autopsy, neuropathological assessment, and research was obtained from all subjects, and all studies were carried out under the ethical approval of the tissue bank. Full consent was provided for use of fixed sections from the US FTD-ALS patient harbouring an Annexin A11 G38R mutation.

Immunohistochemistry (IHC)

Slides initially underwent 2-minute de-waxing washes in two consecutive Xylene filled tanks, followed by two washes in 99% Industrial Methylated Spirit (IMS) and one wash in 95% IMS, all for 2 minutes each. After these initial washes, sections were placed in a tank containing methanol and 30% aqueous hydrogen peroxide solution for 30 minutes. Slides were then washed in running water for approximately 5 minutes, before being moved into distilled water for a rinse and undergoing a microwave-enhanced optimized protocol to open up antigen epitopes. Briefly, slides were incubated in Citrate buffer (0.01M, pH 6.0) and microwaved at "high" setting for 6 minutes, once, then at "simmer" setting for 8 minutes, twice. After being cooled off under tap water for approximately 5 minutes, slides were rinsed twice in Tris Buffer Saline, 5 minutes each. A hydrophobic wax ring was then drawn around each sample of tissue and slides were incubated in a blocking solution (Normal Horse Serum, Immpress IgG Polymer Kit, Vector Laboratories), 250ul of solution each, for 20 minutes at room temperature (RT). Slides were then drained and incubated in primary antibodies (primary antibodies were diluted in Animal-Free Blocker and Diluent, R.T.U., SP-5035, Vector Laboratories) for 1 hour at RT. Samples were washed twice in Tris Buffer Saline (TBS, 1X) for 5 minutes each, before being incubated in secondary antibodies (ImmPRESS, Horse Anti-Mouse or Horse Anti-Rabbit, IgG Polymer Kit, Peroxidase, MP-7402, Vector Laboratories) for 30 minutes at RT. Slides were washed again twice in TBS 1X, for 5 minutes each, and were then incubated in DAB (3,3′-

Diaminobenzidine Enhanced Liquid Substrate System tetrahydrochloride, D3939, Sigma Aldrich) solution. Samples were then moved to running water and then counterstained with haematoxylin for 10 seconds, before being moved back under running water. Slides were dipped in Acid Alcohol (1%, 200 uL 1M Hydrochloric Acid in 2000 mL 70% IMS) for a few seconds and then rinsed under running water and finally into distilled water. Samples were then sequentially washed in the following tanks for 2 minutes each: 70% IMS, 95% IMS, 2x 99% IMS, and four mounting Xylene tanks. Slides were then mounted using DPX-new mounting medium (1.00579.0500, Sigma Aldrich) and glass coverslips (No 1, ECN 631-1573, VWR), and allowed to set at RT.

LMNB2 Quantification

Using a Olympus BX51 microscope at x40 magnification sequential images of the anterior horn regions from spinal cord sections stained with antibodies against Lamin B2 were photographed (Infinity X camera, Infinity Capture Software). This gave a photographic area of 0.0858 mm^2. The number of definite neuronal cells with nuclear staining were counted in this area, together with the number of neurons with full nuclear staining opacity with the Lamin B2 antibody. Evidence of only nuclear folds or crinkles was not accepted as opacity. The ratio of the number of neurons with nuclear opacity to total number of neurons with nuclear staining were calculated per field. Apart from the individual Annexin A11 mutation cases the results were pooled for individual cases and between groups of cases. Apart from the individual Annexin A11 mutation cases there were a minimum of 2 cases per group and a minimum of 15 counts/ratios per group. The Kruskal-Wallis test (followed by post-hoc Dunn's test and Bonferroni correction) for multiple comparisons of the ratios was applied (https://www.statskingdom.com/) with a significance level of 0.05.

There are a number of limitations with this method. Firstly, it relies on limited archival tissue which was by its very nature available from different regions of the cord in each case, and whilst an attempt for consistency was made using thoracic cord whenever possible, this was not always achievable. Furthermore, the results may become skewed because the severity of the disease in particular cases may result in fewer observable neurons. This was attempted to be offset by calculating ratios rather than absolute numbers. Finally, by pooling results from cases it is possible that the effects in individual cases are muted somewhat. However, even with these caveats the results give some indication as to possible differences in nuclear function between Annexin mutation ALS cases and other fALS and SALS cases.

Immunofluorescence

Similarly to the IHC protocol described in the previous paragraph, samples underwent the following washes: 2x in de-waxing xylene tanks, 2x in 99% IMS, and 1x in 95% IMS, all for 2 minutes each. Slides were then rinsed under running water, then moved into distilled water, and finally they underwent the microwave-enhanced optimized protocol to open up antigen epitopes, as outlined in the previous section. After washing the samples under running water, slides were further washed twice in Phosphate Buffer Saline (PBS, 1X), before wax rings were applied and blocking solution was added (Normal Goat Serum, ab7481, Abcam, 1:10 dilution in PBS 1X, ~250uL per slide) and left to incubate for 45 minutes. Slides were then incubated with the chosen primary antibodies for 1 hour at 37°C, before undergoing 2x PBS (1X) washes for 5 minutes each and being incubated in secondary Alexa Fluor antibodies for an additional 45 minutes. From this point onwards slides were kept in the dark as much as possible. Sections were washed 2x PBS (1X) for 5 minutes each before being incubated with a 1X quenching

buffer solution (True Black Plus Lipofuscin Autofluorescence Quencher, 40X in DMSO, 23014, Biotium) for 8 minutes at RT. Once again, samples were washed 2x PBS (1X) for 5 minutes each and they were then incubated with DAPI for 10 minutes at RT. Finally, samples were mounted with 2/3 drops of mounting medium (EverBrite Hardset Mounting Medium, 23003, Biotium) using 1.5H glass coverslips (VWR).

Antibodies

Primary and Secondary Antibodies	Species	IHC Dilution	IF Dilution	Manufacturer	Code
LMNB2	Mouse	1:200	1:200	Santa-Cruz	SC377379
Annexin A11	Rabbit	1:100	1:100	Proteintech	10479-2-AP
MAP2	Chicken	-	1:5000	Abcam	ab5392
ChAT	Rabbit	-	1:200	Abcam	ab178850
Alexa 488	Goat anti-Rabbit	-	1:1,000	Thermofisher	ab150077
Alexa 568	Goat anti-Mouse	-	1:1,000	Thermofisher	ab175473
Alexa 647	Goat anti-Chicken	-	1:1,000	Thermofisher	ab150171

Microscopy

Immunofluorescent post-mortem spinal cord and motor cortex sections were imaged on a Nikon Upright Ni-E with A1R confocal optics, through a 60x oil 1.4NA objective.

Western Blotting

50mg of frozen post-mortem motor cortex from the patient harbouring the Annexin A11 D40G mutation and age matched controls were used to make lysates in RIPA buffer (50mg in 1ml of RIPA). 30ug of protein lysate was run on a 10% Bis/Tris gel and transferred to a nitrocellulose membrane and probed with Rabbit Lamin B2 antibody (Abcam, cat. no. AB151735) at 1/250 and mouse GAPDH (Sigma, cat. no G8795) at 1/2000.

Statistics

For the LMNB2 quantification by DAB staining, a one-way ANOVA test was conducted using the Kruskal-Wallis test (followed by post-hoc Dunn's test and Bonferroni correction).

Supplementary Figure 1

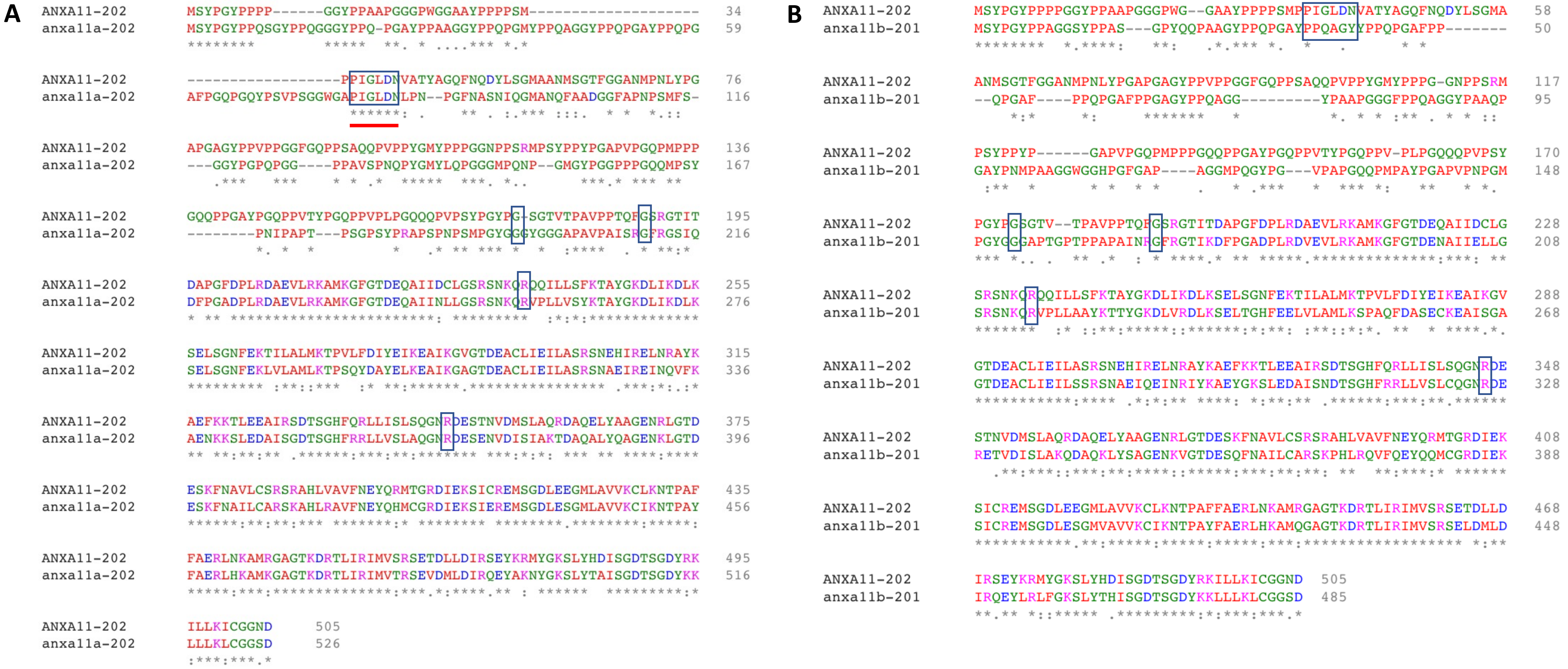

Supplementary Figure 1. Clustal Omega alignment of Annexin A11 protein sequences from human (homo sapiens), and zebrafish Annexin A11a and Annexin A11b orthologues (Danio rerio) (A) Alignment of human Annexin A11 (ENST00000372231.7) with zebrafish Annexin A11a (ENSDART00000101775.5) showing the conserved alpha-helical (AH1) underlined in red. Within this region are the boxed G38 and D40 human residues that are most mutated in ALS+/-FTD cases (boxed in blue). All the mutations identified in our pilot study (Smith et.al Sci Trans Med, 2017) are fully conserved in Zebrafish Annexin A11a (G175R, G189E, R235Q and R346C) (B). Alignment of Human Annexin A11 with zebrafish Annexin A11b (Ensembl Transcript ENSDARG00000002632), with only human residue P36 conserved in zebrafish and a putatively absent conserved alpha helical (AH1) region. N-terminal mutations are not conserved however, C-terminal mutations (like Annexin A11a) retain their conservation.

Supplementary Figure 2

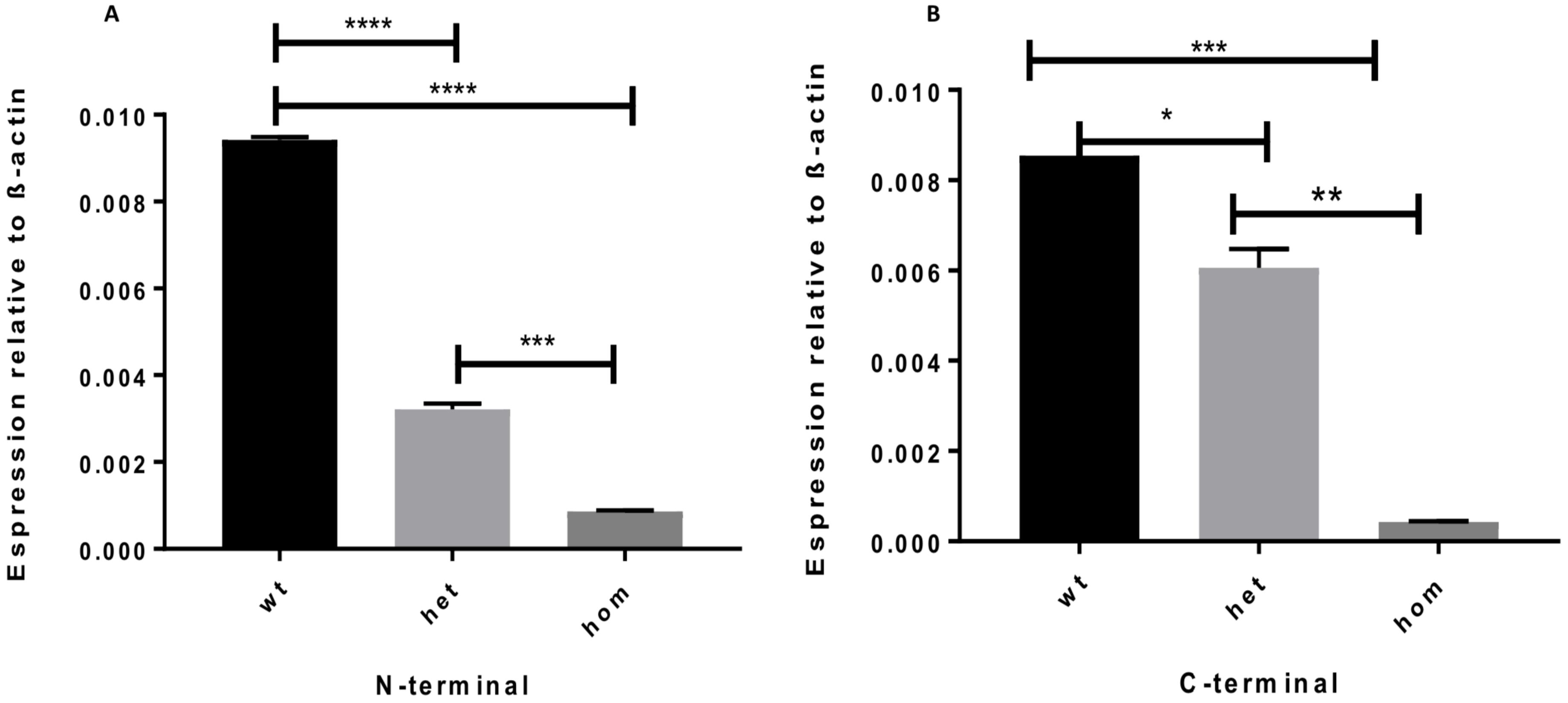

Supplementary Figure 2. Validation of CRISPR/cas9 Annexin A11a exon4 knockout in F3 larvae. (**A-B**) The results from comparative gene expression analysis experiments using cDNAs from 48hpf homozygous and their wildtype and heterozygous siblings are shown. The chart summarises the results for 2 qPCR experiments (n=2).

Supplementary Figure 3

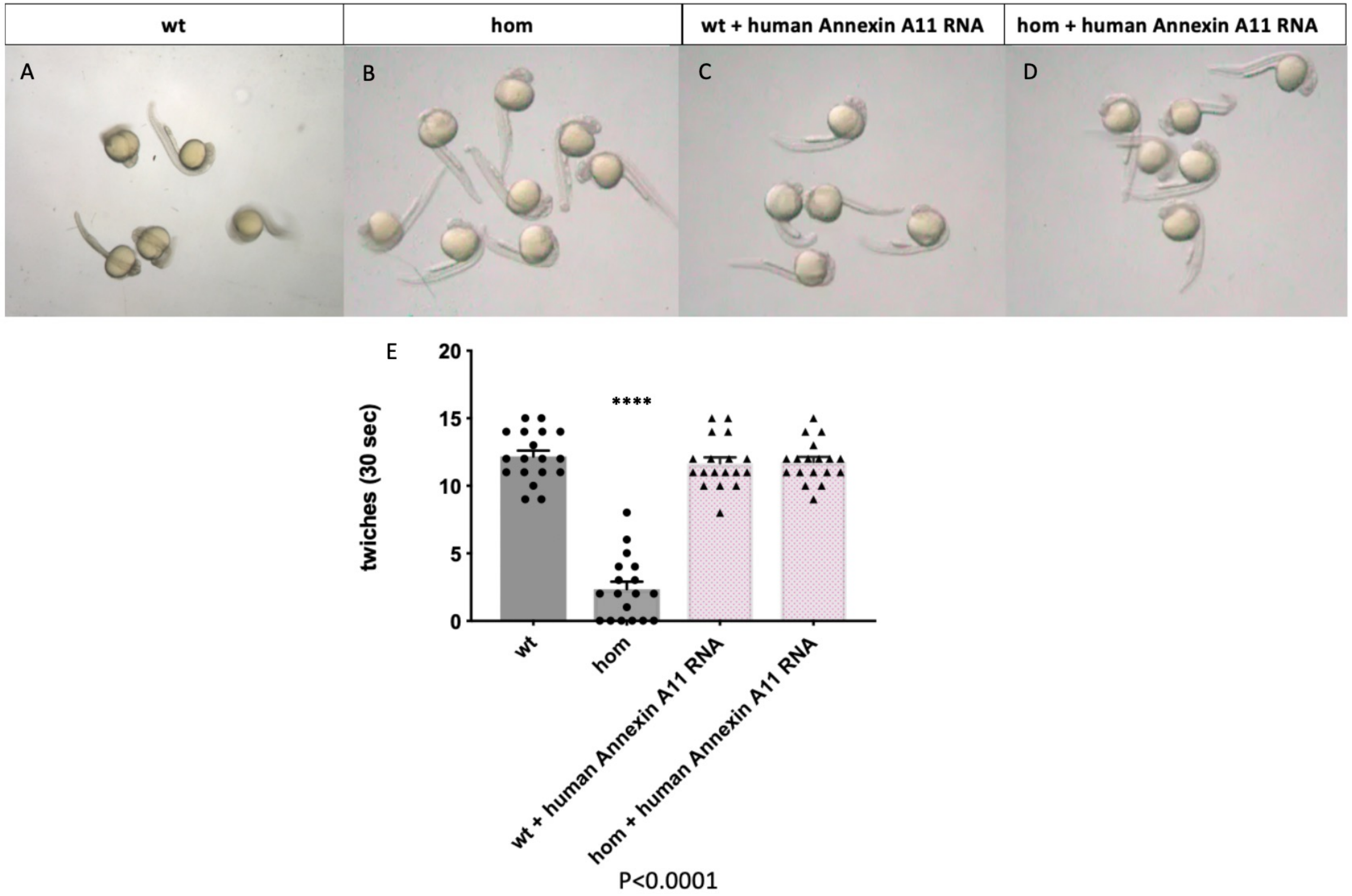

Supplementary Figure 3. Investigating the early motor behaviour in the Annexin A11a zebrafish loss of function model. Panel A and B comparison between WT (A) and HOM (B) zebrafish, at 24hpf homozygous. B Hom lack of spontaneous tail movement, that is present in WT (A). The Tail twitches is completely restored by the injection of Human AnnexinA11 in Hom (D), WT injected with Human WT-Annexin A11-eGFP (C) shows a similar twitches pattern of uninjected WT siblings (A and C). Graph in E shows the quantification of the number of twitches per 30 sec between the 4 groups, showing that the homozygous number is statistically significantly smaller than that of wild-type and this is rescued after the Human wt Annexin A11 RNA injection in Hom with no interference with the ability of WT injected with the human version on the performance of the task (total of 18 embryos were observed for each category). **** $P < 0.0001$ One Way ANOVA with a post-hoc Tukey's multiple comparison test.

Supplementary Figure 4

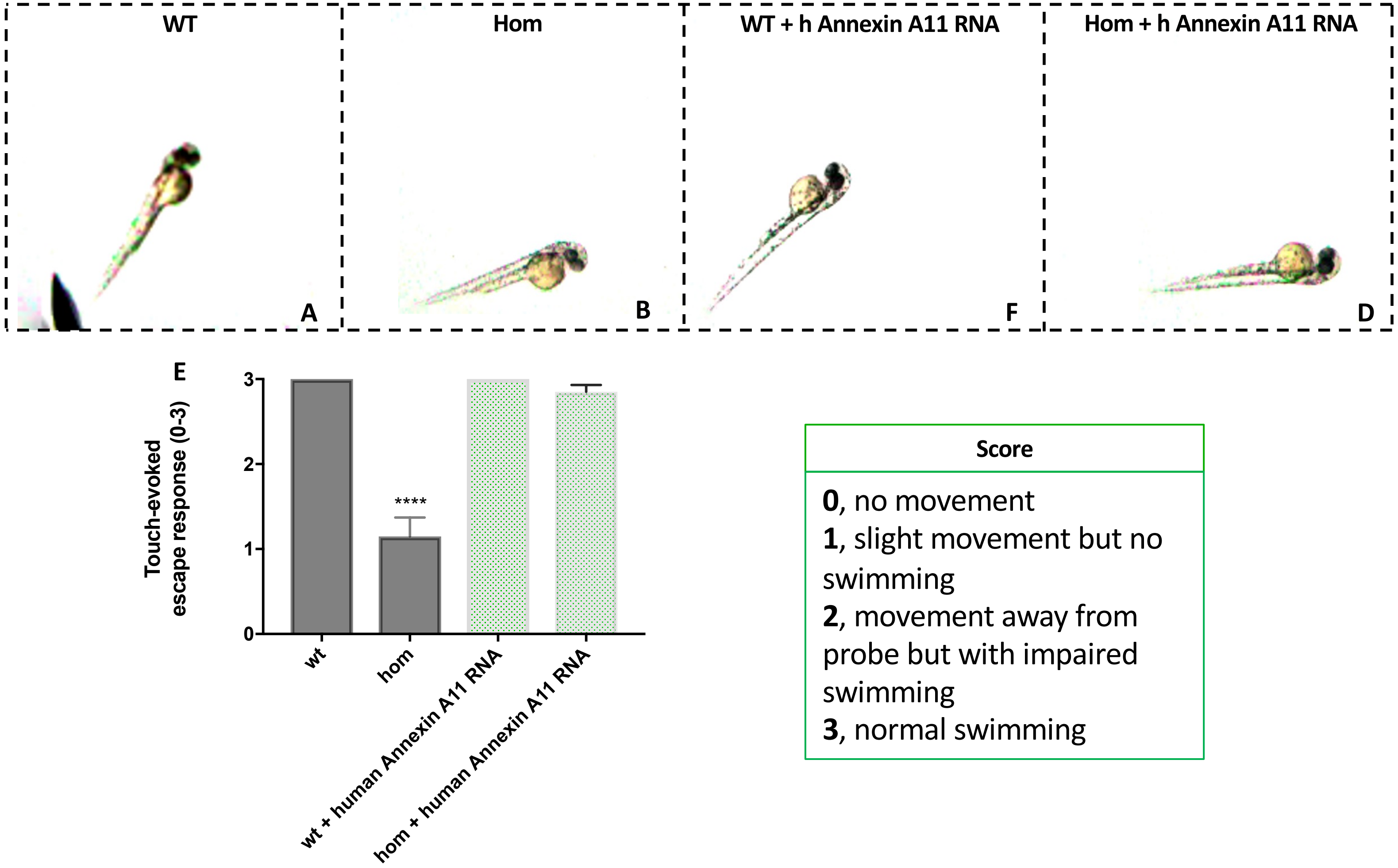

Supplementary Figure 4. Touch- evoked escape response assays in 48hpf Zebrafish. A-D Image and videos (Supplementary data Movies 8-11) representing the swim response comparison between WT (A), homozygous (B) and WT and homozygous injected with human Annexin A11 -GFP (C-D) during the touch- evoked escape response assays (TEER). Briefly, 48hpf zebrafish embryos are gently touched on the tail with a needle and their escape behaviour is assessed and recorded. (E) Quantitative analyses of TEER. Annexin A11a homozygous showed a significant decrease in the movement away from the probe as demonstrated by the scored achieved when compared with all the groups, a behaviour that was restored by the Human WT Annexin A11-eGFP RNA. WT injected with Human WT Annexin A11-eGFP RNA embryos did not differ from the WT un-injected embryos in their task performance (One-way ANOVA ****p<0, 0001; n=20 each group, 3 biological replicates. F scoring system using for the performance evaluation.

Supplementary Figure 5

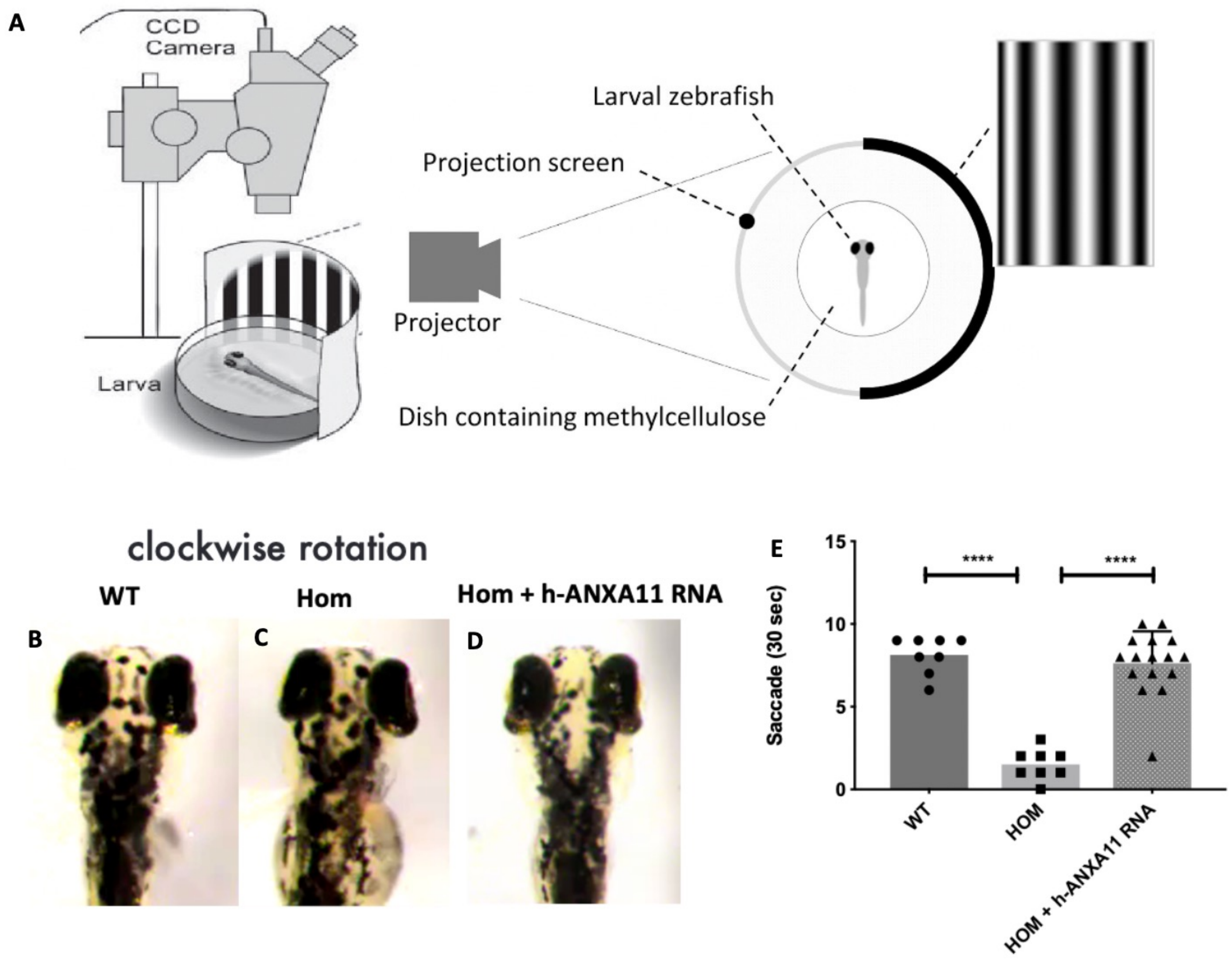

Supplementary Figure 5. OKR of 5dpf Larval Zebrafish. Panel A shows the OKR device which consists of a 14 cm diameter rotating drum and microscope with adjustable light intensity settings. A camera provides a live feed on an adjacent monitor. The rotating drum has various speed settings in both clockwise and counter-clockwise directions and interchangeable spatial frequency gratings are inserted and removed as needed. 5 dpf Larvae are secured and placed in 3% methylcellulose solution while keep alive for a long time in the centre of the rotating drum (Figure sourced from both Scheetz S et.al J. Neurosci. Methods, 2018, 293: 329–337 and Rinner O et.al Investigative Ophthalmology & Visual Science, 2005 (46), 137-142). B-C-D Representative images of 3 larvae (WT and Hom and rescued Hom, Supplementary data Movie 12-14). E. Quantitative analysis of saccade number per 30 sec. (wt n=8, hom=8, rescued hom= 16, 2 biological replicates. **** $P < 0.0001$, One Way ANOVA with a post-hoc Tukey's multiple comparison test).

Supplementary Figure 6

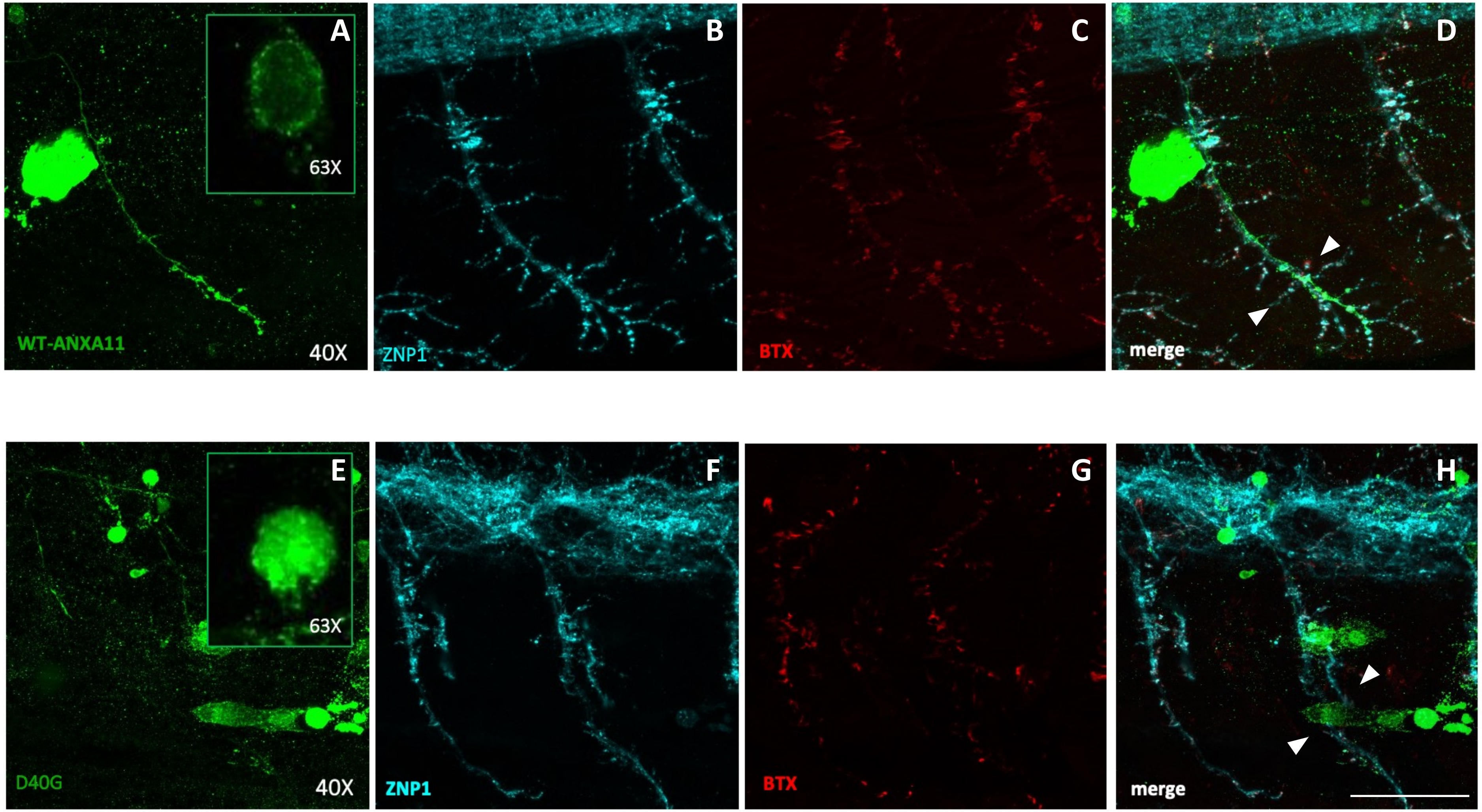

Supplementary Figure 6. **Abnormal NMJs and nuclear accumulation of Annexin A11 is observed in D40G mutant zebrafish embryos**. (A-H) Confocal images of zebrafish trunk, lateral view, anterior to the right, imaged at 48hpf after microinjection with WT-Annexin A11-e-GFP (A-D) and D40G-Annexin A11-e-GFP (E-H) mutant plasmids labelled with GFP (green; A and E), ZNP1 (cyan B and F), α-bungarotoxin (red; C and G); merge D and H; a and e inset of WT-Annexin A11-e-GFP and D40G-Annexin A11-e-GFP nuclei respectively. NMJs' are indicated by white arrows. Inset a and b show the different distribution of WT-Annexin A11-e-GFP (a) and D40G -Annexin A11-e-GFP (b) localized predominantly in the nuclear envelope for WT but distributed and aggregating within the nucleoplasm for the D40G variant (single plane, 63X). Max projections, LSM 800 Confocal using a 40X oil objective. Scale bar = 100µm

Supplementary Figure 7

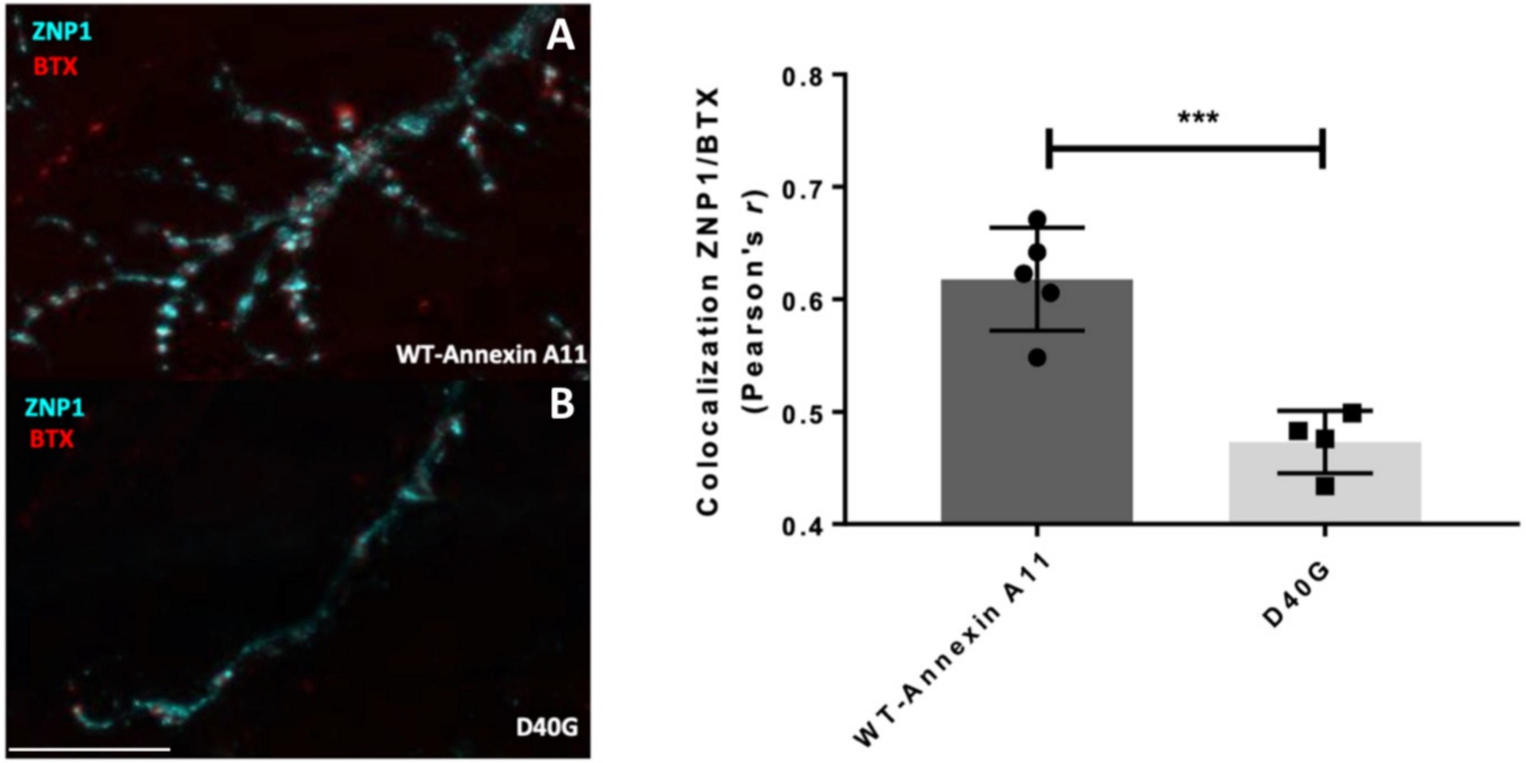

Supplementary Figure 7. NMJs quantification: (A) Whole-mount antibody labelling with ZNP1 2 (cyan) for pre-synaptic vesicles and α BTX, (red) for post-synaptic terminals of WT-Annexin A11-eGFP and (B) p.D40G-Annexin A11-eGFP injected embryos, 48hph. Max projections, LSM 800 Confocal using a 40X oil objective. Scale bar = 100µm. (C) Quantitation of NMJ colocalization of D40G- Annexin A11-eGFP injected embryos in comparison to WT-Annexin A11-eGFP. NMJs were examined in trunk sections by immunolabeling with antibodies to ZNP1 and α-BTX using the Pearson’s correlation coefficient in Image J (***P<0.001, GraphPad Prism 8 using a student’s T-test; n=5 WT-annexin A11 and n=4 D40G-Annexin A11).

Supplementary Figure 8

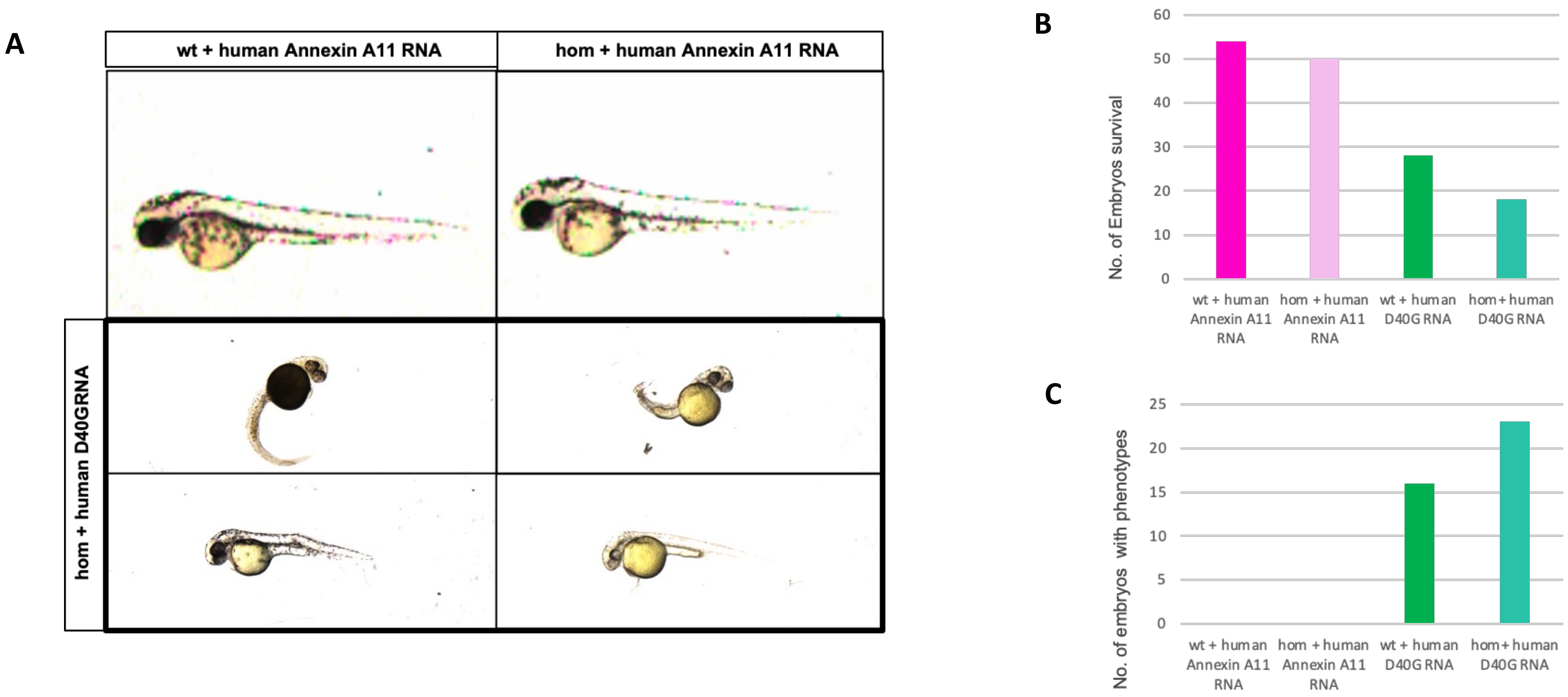

Supplementary Figure 8. Effect of the WT *Annexin A11-eGFP* and *D40G-Annexin A11* RNA (48hpf) on Homozygous embryos. **A**-**B** Representative images of a WT embryo injected with Human WT *Annexin A11-eGFP* RNA (**A**) with no phenotype defects at 48hpf, and a Homozygous embryo injected with Human WT *Annexin A11-eGFP* RNA and displaying a rescued phenotype. (n=120 each group). The D40G mRNA did not rescue and replicated the phenotype observed in non-transgenic larvae mosaically expressing human Annexin A11 D40G DNA

B Average number of embryos WT and Hom embryos which survived post WT *Annexin A11-eGFP* RNA injection (WT=53; Hom=50) and WT and Hom embryos after *D40G-Annexin A11-eGFP* RNA injection. **C** Average number of phenotypes associated post injection of WT *Annexin A11-eGFP* RNA in WT and Hom and after *D40G-Annexin A11-eGFP,* those who survive showed an increased phenotype within the viable embryos in both WT and Hom embryos, however loss of endogenous Annexin a11 is associated with a more severe phenotype. 3 biological replicates.

Supplementary Figure 9

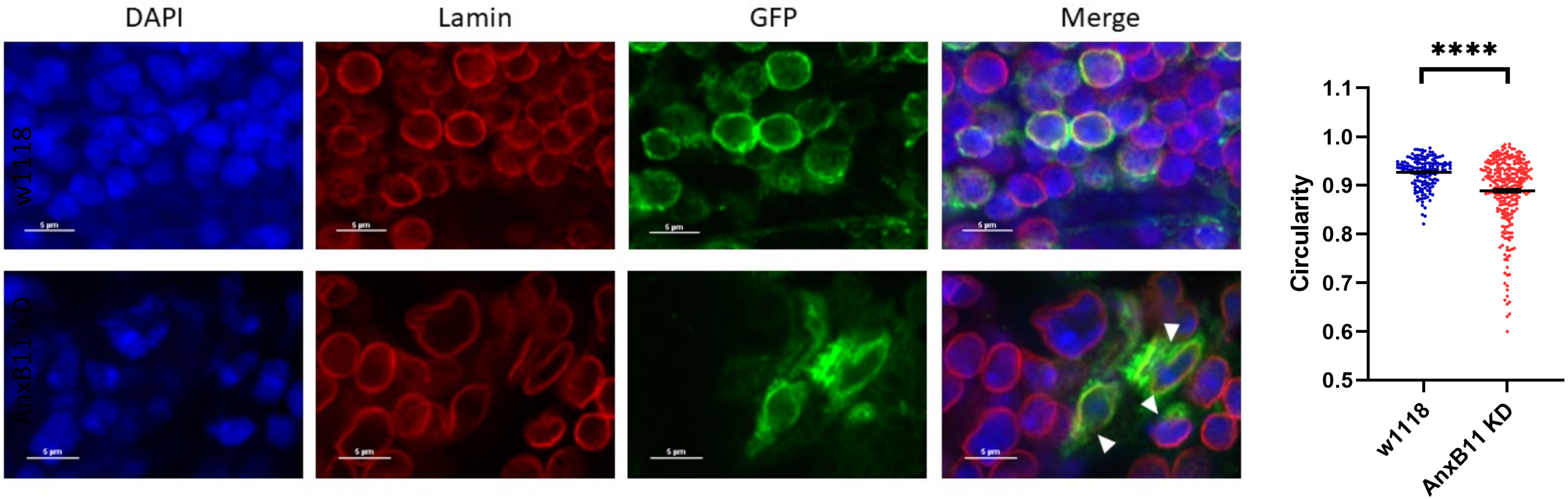

Supplementary Figure 9. Loss of nuclear integrity in AnxB11 knockdown. Drosophila AnxB11 knockdown (29693 RNAi line) and w1118 as a control using OK371, CD8-GFP. Adult flies were aged at 29°C for 22 days. Female flies were dissected and whole brains were incubated with an anti-GFP polyclonal Rabbit antibody (Invitrogen, #A-11122) at a dilution of 1:200 and anti-lamin Dm0 Mouse antibody (DSHB, #ADL67.10) at a dilution of 1:500 for immunostaining. Brains were imaged on the confocal inverted Nikon A1R: DAPI labelled nuclei (blue) and Lamin (red) were used to examine the nuclear circularity in Annexin B11-GFP positive cells (green). Scale bar 5µm. White arrow heads show examples of loss of nuclear integrity in GFP+ cells in the RNAi line. Graph shows a significant loss of circularity (NIS Elements, circularity analysis). Bars: mean ± SEM, w1118 (n = 156) , RNAi 29693 (n=279). Unpaired t test ****p<0.0001.

Supplementary Figure 10

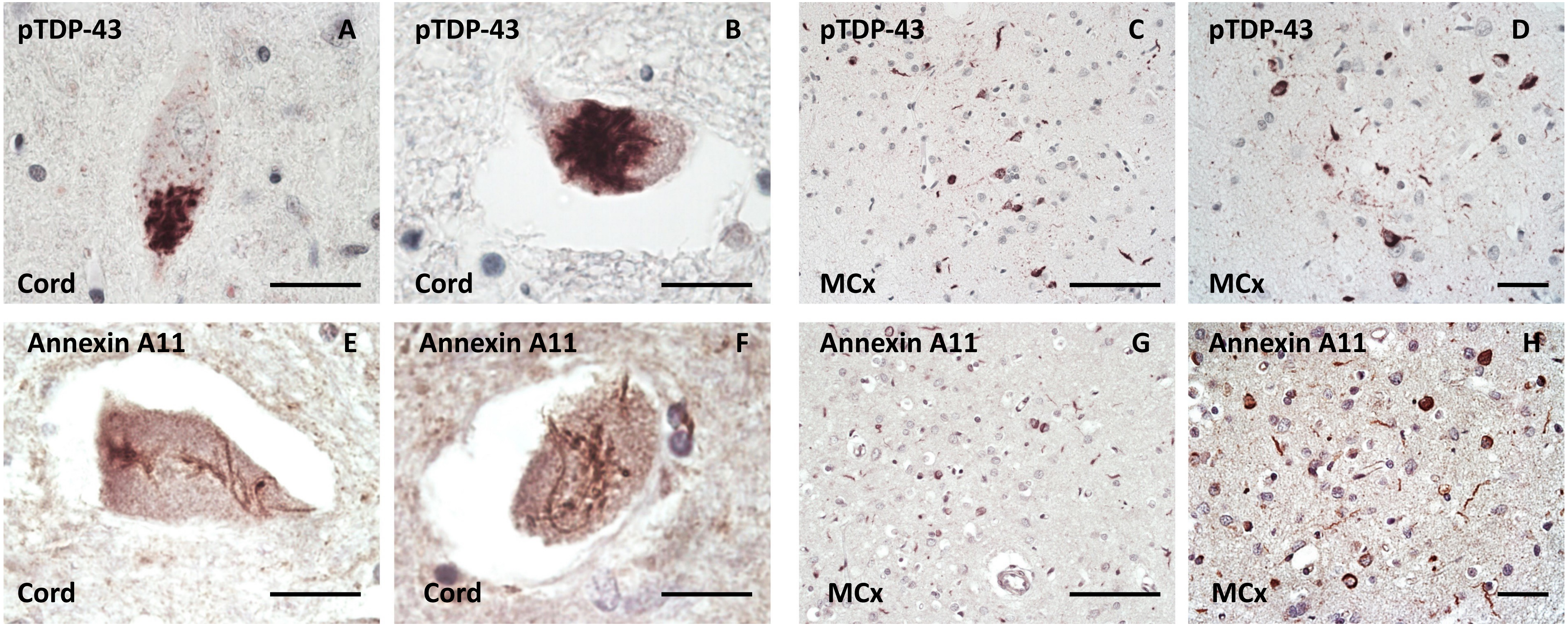

Supplementary Figure 10. Immunohistochemistry on the G38R Annexin A11 mutation case. A-D pTDP-43 immunohistochemistry. E-H Annexin A11 immunohistochemistry. A-B; E-F: Anterior horn neurons from spinal cord. C-D; G-H: Motor Cortex. A-D : There is extensive immunopositivity for pTDP-43 in the anterior horn neurons (A,B) and motor cortex. (C,D). E-H: There are occasional skein-like Annexin A11 immuno-positive neuronal cytoplasmic inclusions (NCIs) in the cord (E,F) and abundant Annexin A11 immuno-positive NCIs and neurites in the motor cortex (G,H). Scale bars A-B, E-F – 50 µm. C,G-600 µm, D,H-200 µm.

Supplementary Figure 11

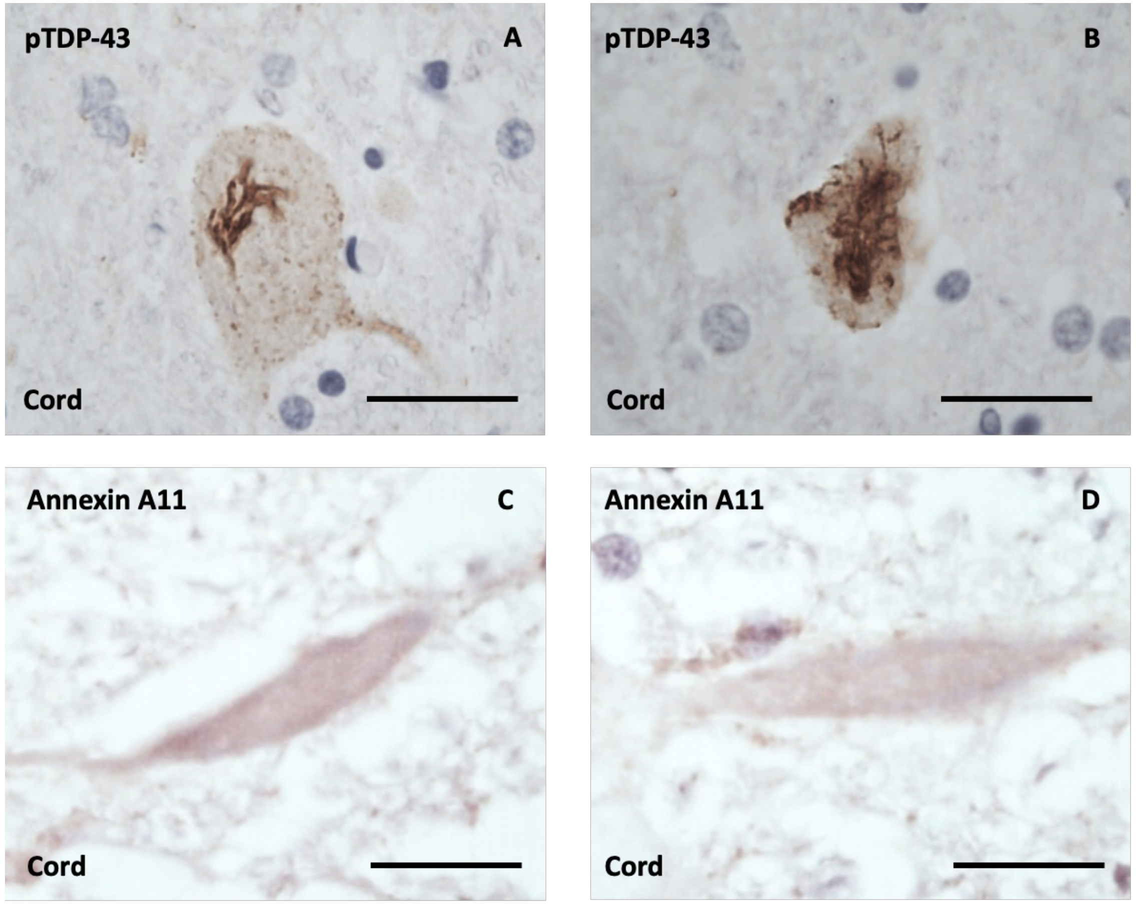

Supplementary Figure 11. pTDP43 and Annexin A11 immunohistochemistry of an Annexin A11 p.R235Q case. Immunohistochemistry on the R235Q ANXA 11 mutation case. A, B, pTDP-43 immunohistochemistry. C,D Annexin A11 (ANXA-11) immunohistochemistry. There is extensive immunopositivity for pTDP-43 in the anterior spinal cord neurons (A,B), but negativity for ANXA-11 in the neurons (C,D). Scale bars 50 µm.

Supplementary Figure 12

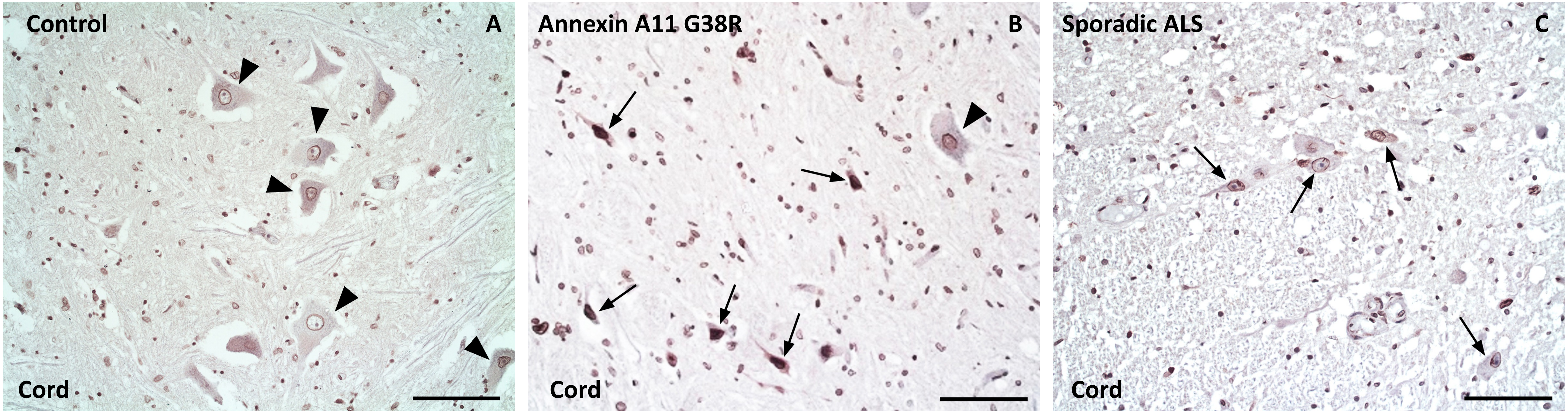

Supplementary Figure 12. Lamin B2 immunohistochemistry. Comparing low power views from the anterior horn of a control case, the G38R mutation case, and a sporadic ALS case (SALS) A. A low power view from anterior horn of spinal cord of a control case stained for Lamin B2 revealing the preserved motor neurons with normal staining nuclear membranes (arrowheads). (Scale bar-200 μm). B. A low power view from the anterior horn of the spinal cord of the ALS-FTD case harbouring an Annexin A11 G38R mutation. Lamin B2 staining illustrates numerous atrophic neurons with diffuse nucleoplasmic staining (arrows) compared with the normal staining nuclear membrane in a preserved neuron (arrowhead). (Scale bar-200 μm).C. A low power view from the anterior horn of spinal cord of a sporadic ALS (SALS) case stained for Lamin B2 revealing the atrophic motor neurons but with normal (non-nucleoplasmic) staining nuclear membranes (arrows). (Scale bar-200 μm).

Supplementary Figure 13

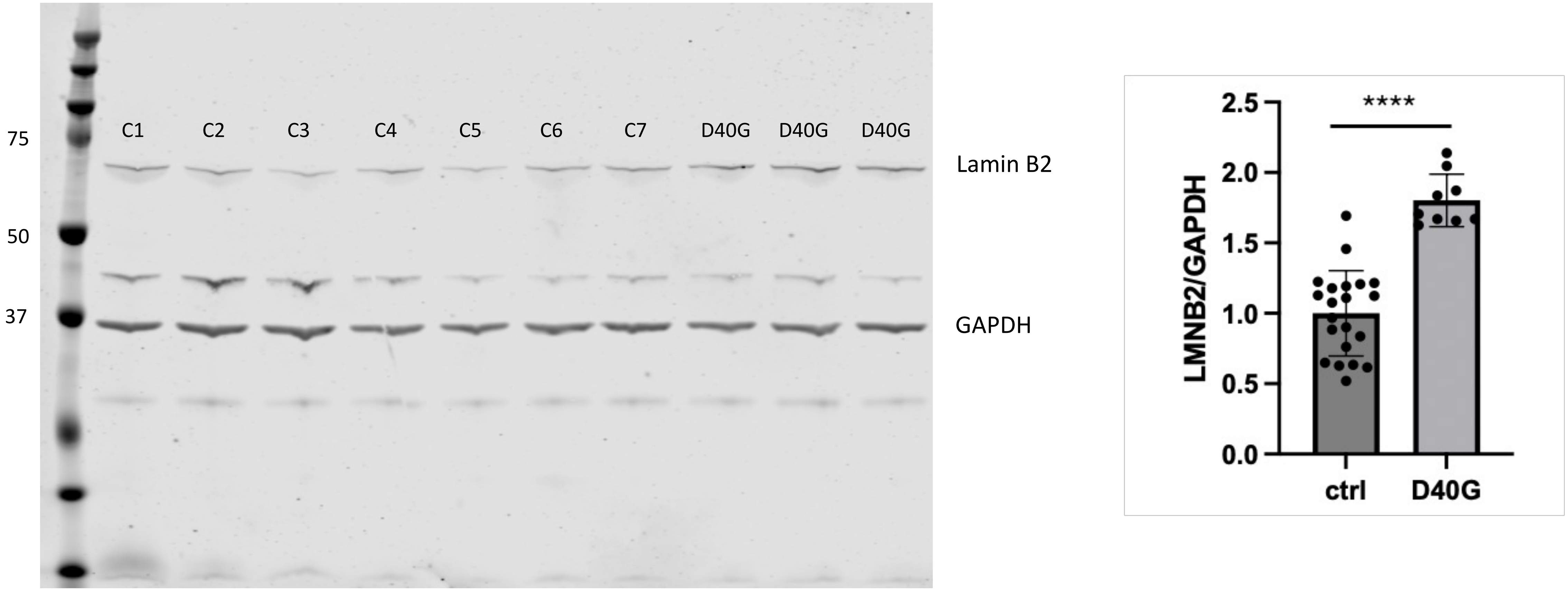

Supplementary Figure 13. Western blot of Lamin B2 expression from motor cortex of Annexin A11 D40G patient. 30ug of whole motor cortex lysate run on a nitrocellulose membrane was stained for Lamin B2 comparing protein expression of D40G (n=3) and control individuals (n=7). Relative to GAPDH expression, Lamin B2 expression is ~1.8 fold higher in the D40G case to controls (unpaired t-test, p<0.0001). Non-specific bands for Lamin B2 and GAPDH were observed at ~40kDa and ~28kDA respectively but were not significantly different in intensity between controls and the D40G case (p=0.41).
